# High throughput SNP discovery and genotyping in grapevine (*Vitis vinifera *L.) by combining a re-sequencing approach and SNPlex technology

**DOI:** 10.1186/1471-2164-8-424

**Published:** 2007-11-19

**Authors:** Diego Lijavetzky, José Antonio Cabezas, Ana Ibáñez, Virginia Rodríguez, José M Martínez-Zapater

**Affiliations:** 1 Departamento de Genética Molecular de Plantas, Centro Nacional de Biotecnología, Consejo Superior de Investigaciones Científicas (CSIC), C/Darwin 3, 28049 Madrid, Spain

## Abstract

**Background:**

Single-nucleotide polymorphisms (SNPs) are the most abundant type of DNA sequence polymorphisms. Their higher availability and stability when compared to simple sequence repeats (SSRs) provide enhanced possibilities for genetic and breeding applications such as cultivar identification, construction of genetic maps, the assessment of genetic diversity, the detection of genotype/phenotype associations, or marker-assisted breeding. In addition, the efficiency of these activities can be improved thanks to the ease with which SNP genotyping can be automated. Expressed sequence tags (EST) sequencing projects in grapevine are allowing for the *in silico *detection of multiple putative sequence polymorphisms within and among a reduced number of cultivars. In parallel, the sequence of the grapevine cultivar Pinot Noir is also providing thousands of polymorphisms present in this highly heterozygous genome. Still the general application of those SNPs requires further validation since their use could be restricted to those specific genotypes.

**Results:**

In order to develop a large SNP set of wide application in grapevine we followed a systematic re-sequencing approach in a group of 11 grape genotypes corresponding to ancient unrelated cultivars as well as wild plants. Using this approach, we have sequenced 230 gene fragments, what represents the analysis of over 1 Mb of grape DNA sequence. This analysis has allowed the discovery of 1573 SNPs with an average of one SNP every 64 bp (one SNP every 47 bp in non-coding regions and every 69 bp in coding regions). Nucleotide diversity in grape (π = 0.0051) was found to be similar to values observed in highly polymorphic plant species such as maize. The average number of haplotypes per gene sequence was estimated as six, with three haplotypes representing over 83% of the analyzed sequences. Short-range linkage disequilibrium (LD) studies within the analyzed sequences indicate the existence of a rapid decay of LD within the selected grapevine genotypes. To validate the use of the detected polymorphisms in genetic mapping, cultivar identification and genetic diversity studies we have used the SNPlex™ genotyping technology in a sample of grapevine genotypes and segregating progenies.

**Conclusion:**

These results provide accurate values for nucleotide diversity in coding sequences and a first estimate of short-range LD in grapevine. Using SNPlex™ genotyping we have shown the application of a set of discovered SNPs as molecular markers for cultivar identification, linkage mapping and genetic diversity studies. Thus, the combination a highly efficient re-sequencing approach and the SNPlex™ high throughput genotyping technology provide a powerful tool for grapevine genetic analysis.

## Background

Single nucleotide polymorphisms (SNP) and insertions/deletions (INDELs) are the most abundant type of DNA sequence polymorphisms and can be theoretically found within every genomic sequence [1,2]. They can be used as genetic markers for many genetic applications such as cultivar identification, construction of genetic maps, the assessment of genetic diversity, the detection of genotype/phenotype associations, or marker-assisted breeding. [3-5]. Furthermore, the development of high throughput genotyping methods make single nucleotide polymorphisms (SNPs) highly attractive as genetic markers [6].

SNPs are a useful tool to quantify LD. The structure of LD along each particular genome or genomic region affects the resolution of association studies [7,8]. For genomes with a slow LD decay with distance, the whole genome may be scanned to identify regions that are associated with a particular phenotype in an association mapping strategy. However, when LD decays rapidly within short distances only nucleotide variation at selected candidate genes may be tested for association with a phenotypic trait [3,9-11].

In plants, systematic analyses of nucleotide polymorphism have only been approached in a few, well-studied model species such as Arabidopsis, barley, maize [12-15] and a few woody perennials species [16-18]. These studies have been either based on the information generated by EST and whole genome sequence projects in a so called *in sili*co SNP discovery approach [16-19] or derived from large-scale re-sequencing projects developed for Arabidopsis, barley, maize, tomato, or soybean [1,12,13,15,20-22]. Independently of the SNP discovery approach, these studies provide significant information regarding the type and frequency of the observed polymorphisms. The reported nucleotide diversity values (π) and number of segregating sites (θ) ranged from π = 0.0063 and θ = 0.0096 in maize [23] to 5–10-fold lower values in soybean (π = 0.0012, θ = 0.00097, [13]), depending on the analyzed parameter. Maize is a highly polymorphic species presenting SNP frequencies corresponding to one SNP every 60 [15] to 104 bp [23], while self-fertilized species show considerable lower values as in the case of barley (one SNP every 200 bp, [12]), soybean (one SNP every 273 bp, [13]), Arabidopsis (one SNP every 336 bp, [14]) or wheat (one SNP every 540 bp, [19]). Values reported for SNP expected heterozygosity are low, as expected for a bi-allelic marker (ca. 0.30 for maize [15] and wheat [19]), while haplotype expected heterozygosity raises to 0.52 in soybean [13] and 0.56 in maize [15]. Regarding short-range LD, several estimations were reported in crop plants like maize, with contrasting values depending on the type of sample (rapid LD decay when using a diverse germplasm set [23] and slow or no-decay when using inbred lines [15]). Alternatively, outcrossed woody species such as spruce, generally display a rapid decay of LD values [24].

The economic relevance of grapevine (*Vitis vinifera *L.) has prompted a considerable effort in EST sequencing and more than 336789 EST entries are currently found at the National Center for Biotechnology Information (NCBI [25]). Recently, the whole genome sequence of an inbred genotype (PN40024) has being completed by a French-Italian consortium [26] and the results of the sequence of the heterozygous cultivar Pinot Noir are also available in databases (IASMA Genomics [27] and NCBI). The final goal of these sequencing efforts is to understand the genetic and molecular basis of production and quality traits in this species what requires establishing the relationship between nucleotide diversity and phenotypic variation.

The original wild grapevine is a dioecious species and hence an obligate outcrosser while domesticated cultivars are hermaphrodite [28] The domestication process could have involved several independent events and a low number of sexual generations including spontaneous cross hybridizations with wild populations [29]. In agreement with these features the grapevine genome is highly polymorphic and the expectation is that the extent of linkage disequilibrium will be generally low in the short range when a sample of genetically distant genotypes is analyzed. Alternatively, if samples of related cultivars within a given region are considered, the extent of LD could be much higher as a result of common domestication bottlenecks and even close family relationships frequently found among them [28]. Until now, only one report [30] has provided a preliminary picture of the frequency and type of sequence polymorphisms in 25 selected gene sequences (ca. 11.6 kb) characterized in seven *V. vinifera *cultivars and two related *Vitis *species. The conclusions of that report were preliminary for *V. vinifera *and no information was provided on the extent of short range LD.

Our primary goal was to characterize the levels of nucleotide polymorphism in *V. vinifera *and to analyze the extent of short range LD. Furthermore we wanted to develop consistent and useful SNP markers for genetic applications in grapevine. Here we report the frequency of SNP and SNP haplotype diversity in 230 randomly selected DNA gene sequences. These fragments span 100.5 kb of DNA sequence associated to coding regions and were re-sequenced in 11 *V. vinifera *genotypes selected from the cultivated and wild genetic compartments of this species. The results allow us to generate more accurate values for nucleotide diversity in grapevine and provide a first estimate of short-range linkage disequilibrium. Using SNPlex™ genotyping technology we have validated the use of the discovered SNPs as molecular markers for linkage mapping, cultivar identification and genetic diversity studies. Thus, the combination a highly efficient re-sequencing approach and the SNPlex™ high throughput genotyping technology [6] provide a powerful tool for grapevine genetic analysis.

## Results and discussion

### Strategy of SNP discovery in the grapevine genome

To identify SNPs in the grapevine genome we used an SNP discovery approach based on re-sequencing in a selected sample of grapevine genotypes. This sample was chosen to include non related wine and table cultivars of ancient origin as well as wild accessions. Based on the available information, they correspond to different cultivar genetic groups [31] and bear chlorotypes belonging to the four major types described in grapevine [27]. The re-sequencing strategy is the most direct way to identify SNP polymorphisms [1] with demonstrated success in different plant species [1,12,13,15,22,32,33]. PCR primers were designed for 451 randomly selected EST sequences. Out of them 184 primer pairs were discarded due to the lack of amplification in more than three genotypes or, in some cases, to the generation of PCR products longer than 1000 bp probably caused by the amplification of unknown intron sequences within the selected ESTs. The remaining 267 PCR fragments were re-sequenced in the set of 11 genotypes, obtaining high quality sequence data for a total of 230 DNA fragments (>86%) with an average amplicon size of 437 bp (Additional file 1). The remaining 37 PCR fragments, although showing good agarose-gel quality did not yield readable DNA chromatograms for the sequence analysis software. Although SeqScape software is able to detect and analyze heterozygous insertions/deletions (INDELS), this is almost impossible when several heterozygous INDELS are located along the same sequence. Unfortunately, this seems to be frequent within intron regions of a highly heterozygous genome like the grapevine one.

### Nature and frequency of SNPs and INDELs in grapevine

As a whole, 100.5 kb were sequenced for each genotype (more than 1 Mb considering an average of 10 re-sequenced genotypes). From them, 81.4 kb corresponded to coding regions and 19.1 kb to non-coding regions, mostly belonging to intron sequences present in the genome sequences amplified with the EST-based designed primers. The nucleotide variation observed through the analysis of these sequences is summarized in Table 1. A total of 1573 SNPs and 52 INDELs were identified among the average of 10 genotypes sequenced with the number of nucleotide polymorphisms per sequence fragment ranging from 0 to 20.

**Table 1:** Nucleotide and haplotype diversity in grapevine

**Parameter**	**Overall**	**(coding/non-coding)**
Number of fragments	230	
Average sample size^1^	10.0	
Average fragment size, kb	0.437	
Total size of amplicons, kb	100.5	81.4/19.1
Total bases sequenced, kb^2^	~2010	
Number of SNPs	1573	1170/403
Frequency of SNP	1 per 64 bp	1 per 69 bp/1 per 47 bp
Number of indels	52	9/43
Frequency of indels	1 per 1932 bp	1 per 9055 bp/1 per 444 bp
Mean nucleotide diversity (π/θ)	0.0051/0.0046	
Maximum nucleotide diversity (π/θ)	0.0246/0.0173	
Minimum nucleotide diversity (π/θ)	0.0004/0.004	
Mean gene diversity	0.30	0.30/0.30
Mean haplotype diversity	0.64	
Mean Tajima D	0.29	
Mean observed haplotypes	6.6	
Mean expected haplotypes	5.8	

^1^Average number of cultivars analyzed through the 230 fragments. ^2^Estimation based on (Total size of amplicons, kb) × (Average sample size) × (two DNA strands).

The SNP variation corresponded to an average of one SNP every 64 bp. Most of the SNPs were bi-allelic, with only four (0.25%) showing three alleles. Among the detailed nucleotide polymorphisms, 59.3% were due to transitions and 40.7% to transversions. This observed transition/transversion ratio (1.46) is similar to the previously reported for grape (1.56; [30]) and potato (1.5, [34]), and higher than the ratio 0.92 reported for soybean [13]. As would be expected, the frequency of sequence variants was higher in non-coding regions (one every 47 bp) than in coding regions (one every 69 bp). In coding regions, we observed a 1:1 ratio of silent vs. non-silent nucleotide changes, with 16% of the non-silent changes giving rise to non-conservative amino acid changes. The ratio of silent vs. non-silent changes (1:1) is higher than the 0.8:1 reported in grape by Salmaso et al [30] but still lower than what has been observed in other species like spruce (1.5:1; [18]) or Arabidopsis (2:1; [14]). The grapevine increased values of non-silent nucleotide changes could suggest the existence of a reduced selection pressure resulting in a higher protein diversity what could be in the base of its phenotypic variation.

Regarding the 52 INDELs identified, one third (17) could be classified as mono-, di-, tri- and tetranucleotide variants whereas the two other thirds (35) represented variable size INDELs ranging from one to 38 bp. The frequency of detected INDELs (one every 1932 bp) is an underestimation. If we consider the intron-bearing sequences that did not yield readable data, we would expect the frequency of INDELs to be at least five times higher in introns. This underestimation was even higher in a previous report [30], where only two INDELs were detected after the analysis of 11629 bp (1 every 5814 bp). This difference could be attributable to a "sampling effect" of the genotypes used or the sequences analyzed since the mentioned work only represents about 10% of the sequencing effort of the present work (ca 100500 bp, Table 1).

The overall SNP frequency observed (1 every 64 bp) was lower than that described by Salmaso et al. (1 every 47 bp), being the difference attributable to the inclusion of non-*vinifera *species in their study (i.e. *Vitis riparia *and a complex genotype Freiburg 99360 derived from multiple crosses involving wild species such as *V. rupestris *and *V. lincecumii*) [30]. Surprisingly, the frequency of polymorphisms reported was lower in non-coding regions (1 every 57 bp) than in coding regions (1 every 43 bp). In any case, our results agree with those of Salmaso et al. [30] in displaying a high rate of polymorphisms. The values observed in grapevine were within the range of values reported for maize (one SNP every 60 to 104 bp), which is also a highly polymorphic outcrossing species [15,23] and higher than those observed in self-crossing species such as barley [12], soybean [13], wheat [19,35] or Arabidopsis [14,32]. Consistently, nucleotide diversity values observed in grapevine (θ = 0.0046, π = 0.0051) were similar to those observed in maize (θ = 0.0096 [23], π = 0.0063[15]) and ~5-fold higher than those reported for soybean (θ = 0.00097, π = 0.0012, [13]) or human beings (θ = 0.0008, [36]).

### SNP and haplotype diversity

Diversity values (expected heterozygosity) for SNP are generally low due to their bi-allelic nature. In grapevine, SNP diversity values ranged from 0.00 to 0.66 with a mean value of 0.30 (Table 1) which is slightly higher than the mean value reported for maize (0.26; [15]). Grapevine SNP show lower diversity values than SSR (0.65; [37]), and therefore are less informative markers (average polymorphism information content – PIC – for SNPs is 0.25 as compared to 0.60 for microsatellite [37]). This potential drawback of SNP can be overcome either by using larger sets of markers or by considering haplotypes structure for each locus in place of single SNPs. When haplotypes are considered for each locus, the genetic diversity value rises more than 2-fold (0.64), reaching similar values as those reported for grapevine microsatellites (0.65; [37]) and slightly higher than those reported in maize (0.56; [15]). In this context, SNPs can be as informative as multiallelic molecular markers when used as "haplotype tags", that is, several SNPs (usually two to four) that tag all the detected haplotypes in a given locus [1,38].

### Allele distribution and haplotype structure

The allele distribution in the set of cultivars selected for this study was analyzed by calculating the Tajima D statistic, designed to test the neutrality of mutations [39]. Sequence specific Tajima D values ranged from -1.73 to 2.63 with an average of 0.29. Thus, no indication for an overall deviation of this parameter was observed among the 230 analyzed sequences. Only one sequence, annotated as encoding a putative ortholog of one Arabidopsis calcium-transporting ATPase 9, showed a strong positive Tajima D value (2.63; *P *< 0.01) which could suggest the possible existence of balancing selection operating in this locus [40]. In any case, these results should be taken with caution given the reduced sample size analized.

**Figure 1:**
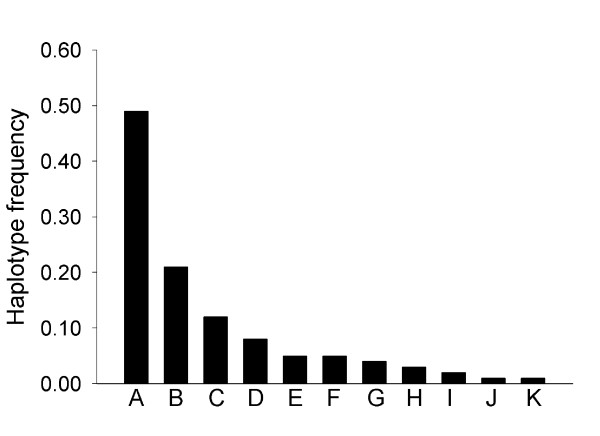
**Distribution of haplotype frequencies for the analyzed sequences**. Mean haplotype frequencies are sorted in decreasing order. Class "A" corresponds to the most frequent haplotype, Class "B" to the second most frequent haplotype in each sequence and so on consecutively.

The number of haplotypes per locus was estimated using the EM algorithm [41]. The average number of haplotypes per sequence was 6.6, with a maximum of 19 and a minimum of 1 (considering haplotype frequency >0.01 and a maximum of 20 SNP polymorphisms as PowerMarker parameters). As displayed in Figure 1, the most common situation was the presence of a major haplotype (average frequency = 0.49), with the average cumulative frequency of the first three haplotypes being 0.83, followed by a series of minor haplotypes. Even though the grape haplotypic parameters presented here could be biased by the chosen cultivars, mean haplotype number and frequency, as well as haplotype frequency distribution, were in agreement with the results reported by Salmaso *et al. *[30]. A similar haplotype distribution has been observed in other species such as maize [15] and barley [12]. The expected mean number of haplotypes per locus was also estimated based on values of nucleotide diversity and recombination using the coalescence theory implemented in DnaSP software [42,43]. The mean number of expected haplotypes (5.8) was similar to the mean number estimated above (6.6). These similar numbers of average haplotypes obtained by both methods together with the absence of bias for the average Tajima's D value could suggest a reduced selection for specific haplotypes within the gene sample analyzed.

### Linkage disequilibrium

**Figure 2:**
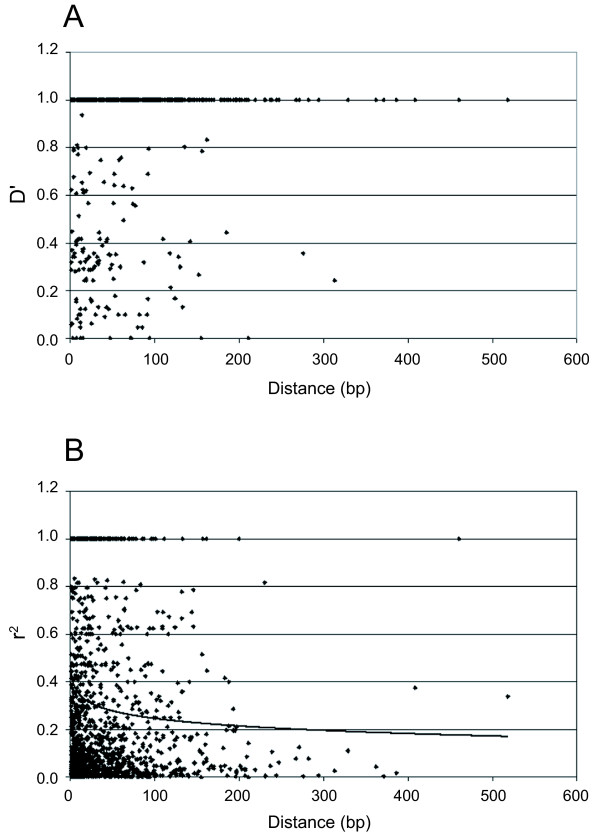
**Linkage disequilibrium decay plot as a function of distance**. Two measures of LD are shown, D' (A) and r^2 ^(B) as a function of distances (bp). Pair-wise LD values between SNP loci corresponding to all sequences fragments presenting at least 2 SNPs were plotted.

In outcrossing species linkage disequilibrium (LD) generally decays rapidly in the absence of selection, which when existing produces locus-specific bottlenecks [8]. To estimate the level of short-range LD in the grapevine genotypes studied, we performed pair-wise analyses in more than 200 loci containing two or more SNPs. For the pair-wise LD analysis we calculated D' and r^2 ^parameters for SNP loci within each sequence. Decay of D' and r^2 ^was observed between 100–200 bp (Figure 2) within this sample. Consistent with these results, a multi-locus LD analysis only detected significant LD (*P *< 0.01) for 4.4% of the within gene pairs of SNP loci. Similar patterns of rapid LD decay were observed in other outcrossing species such maize and woody perennial species like the Norway spruce (*Picea abies*, [24]) or the European aspen (*Populus tremula*, [44]). However, the mating system alone does not always predict the structure of LD since other factors like the sample under analysis or the mating history can affect the LD pattern. This was observed in maize, where short-range LD analysis using an elite germplasm displayed slow or null LD decay [15]. On the other hand, the self-pollinated soybean (*Glycine max*) displayed very low levels of short-range LD [13], probably reflecting the outcross rate of its ancestor *G. soja *(ca. 13%, [8,45]). Only one grapevine gene, *VvMybA1*, responsible for berry color in grapevine [46,47], has previously been evaluated for LD at nucleotide range scale in a selected collection of grapevine cultivars maximized for genetic diversity [48]. In this example, r^2 ^was observed to be close to 0.2 along ca. 700 nucleotides and then rapidly decay [48]. This result is within the range of what we observed in our sample of genotypes and sequences. In contrast, significant LD was reported in grape, at the centiMorgan (cM) scale, when using SSR markers [49], a discrepancy that has been observed in other species such as maize [10] or humans [50].

### SNP genotyping applications

The final goal of the SNP discovery project was to develop molecular markers that could be combined with a high throughput genotyping technology such as SNPlex™ to address different genetic applications [1,2]. The re-sequencing experiments provided the information required to fulfill three important criteria for the selection of SNP to be included in SNPlex™ designs: 1) A clean sequence context (i.e., absence of secondary SNPs surrounding the chosen SNP); 2) A frequent presence of the SNP in populations under study for what we applied the criteria of considering SNPs when present in at least 2 of the "original genotypes" and both strand chromatograms confirmed the polymorphism; 3) Sequence uniqueness (according to the limitations of the grape genome and EST sequence databases). The percentage of the initial submitted SNPlex™ design that produces useful SNPs (known as the Conversion Rate) is dependent on the DNA quality, the validation state of the SNPs, and the presence of genomic repeats. Out of 96 SNPs submitted for validation we succeeded to genotype 80 of them, including one INDEL [(SNP605_120i), Additional file 2]. This conversion rate (83.3%) is within the expected SNPlex™ performance according to manufactures specification (≥ 80%; Product Bulletin "SNPlex™ Genotyping System", [51]). Those 80 SNPs were genotyped in ca. 360 grape genotypes, including accessions and segregating progenies (Additional file 3), with a success rate of 93.5% within the sample. Genotyping errors were estimated by the independent analyses of different plants of the same genotype to be <3 × 10^-4^.

One important feature for the wide application of SNPs in genetic analyses is their Minor Allele Frequency (MAF) value which affects the information provided by the marker in different genetic applications such as linkage and association studies. In general, SNPs with MAF values ≥ 0.05 or 0.10 can be considered as common SNP that are useful in most applications. An analysis of MAF values for the 80 genotypes SNPs in a sample of ~300 *V. vinifera *accessions including a large set of wine and table grape cultivars and wild populations, showed that 80% of them displayed MAF ≥ 0.10, with an average MAF of 0.24 (Table 2). The MAF values observed in this sample of ~300 accessions was correlated (r^2 ^= 0.61) with the MAF values observed in the original sample of 11 genotypes used in the re-sequencing strategy (Additional file 2 and 4). These results support the choice of the genotypes for the re-sequencing approach in the identification of useful common SNPs. Alternatively, SNPs that are specific for a given population can have a high discriminant value to identify the individuals of such population. A partition analysis of SNP frequencies in wild and cultivated table and wine cultivars of *V. vinifera*, showed that none of the selected SNPs were specific of any of these groups (Additional file 5). However, their frequency in each group of genotypes (wild, table and wine) was significantly different to the overall frequency (χ^2^, *P *< 0.01, 1 d.f.) for 41%, 24% and 28% of the SNPs respectively (Additional file 5). These results support the utility of these SNPs for genetic diversity applications.

**Table 2:** Distribution of SNP MAF in grapevine genotypes^1^

**MAF classes**					
*Mean*	0.50-0.40	0.39-0.30	0.29-0.20	0.19-0.10	<0.10
*0.24*	15%	24%	11%	30%	20%

^1^Values are calculated for 80 SNP loci genotyped in 295 accessions.

**Figure 3:**
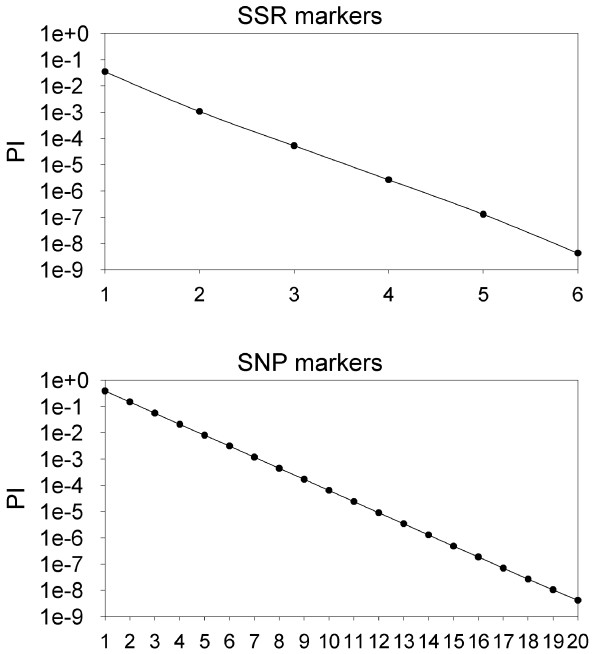
**Probability of identity (PI) values for SSR and SNP markers**. Determination of the number of markers needed to reach a discriminant PI value for cultivar identification (dotted line, ~4 × 10^-9^) with SSR (A) and SNP (B). Y axis is represented on logarithmic scale.

Cultivar identification is an important issue in grapevine where the estimation is that there are over 10000 vegetatively propagated genotypes, frequently confused due to the existence of multiple synonyms and homonyms [52]. Currently, six SSR loci are considered to be sufficient for genetic identifications of most cultivars [53], with a cumulative probability of identity (PI) of 4.3 × 10^-9^. However, in spite of all the effort dedicated to SSR genotyping and standardization of allele sizes and genotypes [53], there are still frequent problems of allele identification among laboratories using different DNA fragment separation technologies. Moreover, SSR genotyping is difficult to multiplex. Given the low PIC of SNPs compared to SSRs a higher number of SNP markers are required to reach similar (PI) in genetic identification. In fact, to reach a similar PI as the six SSR markers currently in use, we estimated that 20 SNP with MAF ≥ 0.30 will be required (Figure 3). This can be easily approached given the facility for multiplexing provided by different SNP genotyping technologies. Furthermore, the bi-allelic nature of SNPs could enormously facilitate the accuracy and repeatability of SNP genotypes avoiding the differences in allelic assignment among laboratories mentioned above [53]. In this context, our expectation is that a panel of 48 validated SNPs and selected for MAF ≥ 0.30 and homogeneous distribution along the grapevine genome could definitively solve most genotyping problems in this species.

Linkage mapping has become a common approach to determine the genetic basis of qualitative and quantitative traits in grapevine. The heterozygous nature of grape cultivars, makes linkage mapping to be performed in F_1 _populations and maps are constructed for each of the parental genotypes using a pseudo test cross strategy [54-59]. For this reason, only markers that are heterozygous in any of the parental genotypes can be placed in the genetic map. The large number of SSR markers developed in grapevine and their multi-allelic nature facilitates the task of building framework maps. However, the difficulties for multiplexing and automatization of SSR genotyping makes the process tedious and time consuming. The usefulness of SNP markers in linkage analyses is related to their MAF values. We analyzed the segregation of the 80 genotyped SNPs in four available mapping populations (Additional file 3). The results showed that SNPs segregating in all four populations (8%) displayed the highest MAF values (X¯
 MathType@MTEF@5@5@+=feaafiart1ev1aaatCvAUfKttLearuWrP9MDH5MBPbIqV92AaeXatLxBI9gBaebbnrfifHhDYfgasaacPC6xNi=xH8viVGI8Gi=hEeeu0xXdbba9frFj0xb9qqpG0dXdb9aspeI8k8fiI+fsY=rqGqVepae9pg0db9vqaiVgFr0xfr=xfr=xc9adbaqaaeGacaGaaiaabeqaaeqabiWaaaGcbaGafmiwaGLbaebaaaa@2D24@ = 0.36), while 16% of the tested SNPs with a mean MAF of 0.08 did not segregate in any of them (Table 3). Thus the selection of a large number of informative SNPs (MAF ≥ 0.30 and homogenous distribution along the genome) combined with high multiplex technologies can provide a rapid strategy for linkage map construction. On the other hand, the number of markers that can be mapped in a given segregating population per parental cultivar depends on their heterozygosity. On average, grapevine accessions genotyped for the 80 SNPs were heterozygous at 30% of the loci (Additional file 2). In this way, a multiplex set with 2000 validated SNPs would allow the rapid position of ca. 600 markers per parental map, what, for randomly selected SNPs and a map size of 1500 cM, approximately represents a probability higher than 95% of having a marker every 10 cM.

**Table 3:** Percentage of SNPs segregating in different mapping populations^1^

Number of mapping populations	SNPs	Average MAF value
4	8%	0.36
3	24%	0.31
2	21%	0.28
1	31%	0.17
0	16%	0.08

^1^Based on the segregation of 80 SNPs-

### Re-sequencing versus other SNP discovery approaches

The highly polymorphic nature of the grape genome represents a challenge for the efficient implementation of in-silico SNP discovery approaches, even those based in whole genome sequencing projects [60] or in EST libraries data-mining [16-18,61]. Two genome sequencing projects have been developed in grapevine. The Franco-Italian sequencing project has recently published the sequence of a near-homozygous genotype derived from cultivar Pinot Noir (PN40024) [26]. In addition, the IASMA sequencing project is releasing the sequence of this cultivar [27]. Sequencing one heterozygous cultivar as Pinot Noir, generates a large number of SNPs directly useful in linkage analyses in progenies derived from this cultivar [62] but does not provide information on their MAF and genome sequence context (i.e. presence of secondary SNPs in other cultivars). A similar situation is observed for *in silico *SNP discovery approaches based in EST libraries, such as the public PlantMarkers database [61], since grape EST database is monopolized by cultivar Cabernet Sauvignon (65% of the EST sequences) and in a far second place cultivar Chardonnay (20%) (*Vitis vinifera *UniGene Build #4; [25]). In a small-scale test performed in our lab, only 25% of the higher score SNPs selected from the PlantMarkers database could be validated by a dCAPs strategy [63] (data not shown).

To demonstrate the efficiency of the re-sequencing approach in grapevine SNP discovery we determined the number of SNPs present in 50 random sequenced fragments from Cabernet Sauvignon and Pinot Noir. According to the observed frequency of one SNP every 64 bp (Table 1), we expected 297 SNPs in the ~19000 bp spanned by the 50 fragments. A total of 323 SNPs were observed within the 11 parental cultivars, when only 115 SNPs would have been identified in Cabernet Sauvignon (35%) and 82 SNPs (25%) in Pinot Noir. Furthermore, the information available for SNPs identified through a re-sequence approach in a selected set of genotypes is particularly important when SNPs markers are selected for high-throughput genotyping technologies, since a wrong or incomplete information regarding the SNP relative frequency or the presence of secondary SNPs could jeopardize the detection assay [6]. Thus, a re-sequencing approach appears determinant to identify useful SNPs for wide genetic applications. Furthermore, the availability of the whole genome sequence should allow a positional selection of DNA fragments to be re-sequenced, enhancing the usefulness of the discovered SNPs.

## Conclusion

We report here an analysis of nucleotide sequence variation in the grapevine genome based on the scanning of >100 kb of DNA sequence in an average of 10 selected genotypes. The results provide detailed information regarding nucleotide diversity in coding associated regions as well as SNP and haplotype diversity. As expected for a dioecious species, we observe a very rapid decay of short range LD within 100–200 bp. The sequence information generated has been used to develop a SNP discovery approach in grapevine providing SNPs of suitable quality for high throughput genotyping technologies such as SNPlex™. Using this genotyping technology in grapevine we have validated the selected SNPs as molecular markers for genetic diversity, cultivar identification and linkage mapping analyses supporting the choice of a re-sequencing approach as an efficient way to generate high quality molecular markers in grapevine. The SNP markers tested in this work are sufficient to provide multiplex approaches for cultivar genetic identification in grapevine. However, the development of SNP marker sets for linkage analysis will require additional re-sequencing efforts to generate sets of a few thousand, high MAF, SNPs evenly distributed along the genome.

## Methods

### Plant material and genomic DNA isolation

Grapevine (*Vitis vinifera *ssp. *sativa*) genotypes used for SNP discovery were selected to included wine cultivars (Cabernet Sauvignon, Syrah, Pinot Noir, Grenache, Tempranillo, Malvasía de Sitges, Muscat à petits grains blanc), table grape cultivars (Sultanina and Ahmeur Bou Ahmeur) and wild accessions (two genotypes of *Vitis vinifera *ssp. *sylvestris*) from populations sampled in the Iberian Peninsula (they are referred as the "original genotypes" in the text). Additionally, 368 accessions were used for genotyping analysis (see below). These accessions are mostly maintained at the germplasm collection of "El Encín" (IMIDRA, Alcalá de Henares, Madrid, Spain). These accessions included cultivated and wild accessions as well as 53 F_1 _hybrids from four different mapping populations (Cabernet Sauvignon × Monastrell, Dominga × Autumn seedless, Ruby seedless × Moscatuel and Muscat Hamburg × Sugraone). Their name, collection code and main use (either wild accession, wine or table grape cultivars) are listed in Additional file 3. Young leaf samples were used for DNA extraction. Genomic DNA isolation and quantification was performed according the procedures described by Lijavetzky *et al. *[47]. DNAs were sorted in 96-well plates and stored at -20°C.

### Selection of target sequences and primer design

The UniGene database [64] stored at the National Center for Biotechnology Information (NCBI) was the main source of grape EST sequences used for SNP discovery. Sequences corresponding to each EST cluster were downloaded to BioEdit v7.0.5.3 software [65] and re-analyzed by means of the CAP3 program [66]. Target sequence regions of ca. 400 bp were chosen to reduce the effect of unknown introns in the length of the resultant sequence and maximize the chances of obtaining SNP polymorphisms. Those target sequences were used as templates for primer design using Primer3 [67] under the default primer selection conditions. Universal M13 forward and M13 reverse promoter homologous sequences were added to each primer pair to facilitate direct sequencing of the PCR products. Primer sequences are available as supplementary material (Additional file 1).

### PCR amplification, sequencing and SNP discovery

PCR amplifications were performed in 25 μl reactions including 1–10 ng of grape genomic DNA, 1 u of AmpliTaq Gold DNA Polymerase (Applied Biosystems), 1× reaction buffer, 1.5 mM MgCl_2_, 0.2 mM dNTP and 0.2 mM of each primer. Amplifications were done on a GeneAmp PCR System 9700 with 10 min at 95°C followed by 35 cycles of 1 min at 94°C, 1 min at 60°C and 1 min at 72°C, with a final extension cycle of 7 min at 72°C. PCR products were verified by electrophoresis in 1.5% agarose using 0.5× TBE buffer, stained with ethidium bromide and visualized under UV light.

Amplified PCR products (5 μl) were treated with 0.2 μl of ExoSAP-IT reagent (USB Corporation) in a 10 μl final volume. Treated PCR products were sequenced at the Genomic Unit of the Parque Científico de Madrid using Universal M13 forward and M13 reverse primers in an ABI Prism 3730 (Applied Biosystems) DNA sequencer. Base calling, quality trimming and alignment of ABI chromatograms was performed using SeqScape Software v2.5 (Applied Biosystems). Sequence polymorphisms were verified manually with the help of BioEdit v7.0.5.3 software [65]. Identification of coding and non-coding regions was performed by means of the BLASTX program using the NCBI [25] and GENOSCOPE BLAST Server [68].

### Statistical analyses

Estimates of nucleotide polymorphism (*Nucleotide diversity *π, the average number of nucleotide differences per site between two sequences [69], and *Number of segregating sites *θ [70]) were obtained using DnaSP software v.4.10 [43] Gene diversity, often referred to as expected heterozygosity [71] was calculated as 1−∑Pij2
 MathType@MTEF@5@5@+=feaafiart1ev1aaatCvAUfKttLearuWrP9MDH5MBPbIqV92AaeXatLxBI9gBaebbnrfifHhDYfgasaacPC6xNi=xH8viVGI8Gi=hEeeu0xXdbba9frFj0xb9qqpG0dXdb9aspeI8k8fiI+fsY=rqGqVepae9pg0db9vqaiVgFr0xfr=xfr=xc9adbaqaaeGacaGaaiaabeqaaeqabiWaaaGcbaGaeGymaeJaeyOeI0YaaabqaeaacqWGqbaudaqhaaWcbaGaemyAaKMaemOAaOgabaGaeGOmaidaaaqabeqaniabggHiLdaaaa@34B9@, where *P*_*ij *_is the frequency of the *j*th allele for *i*th locus, was calculated by means of PowerMarker V3.25 software [72]. Tajima's D test [39], was used to test the hypothesis that mutations at each locus are selectively neutral. The test is based on the differences between the number of segregating sites and the average number of nucleotide differences and was calculated using DnaSP software v.4.10 [43].

For each target locus the haplotype number and frequency and the expected haplotype heterozygosity were calculated using the EM algorithm [41] implemented in PowerMarker V3.25 software [72]. Estimation of expected number of haplotypes, given the estimated values of π and recombination, using coalescent process simulations, was performed with DnaSP software v.4.10 [43].

Decay of LD with distance in base pairs (bp) between sites within each locus was evaluated by nonlinear regression [73]. Linkage disequilibrium (D' and r^2^) between two loci in the genome and the exact test for multi-locus association were calculated as described by Zaykin *et al. *[74] using PowerMarker V3.25 software [72].

Probability of identity (PI) for SSR and SNP markers was calculated by means of the Multilocus option of the GenAlEx6 software [75].

### Genotyping analysis

Selected SNPs and INDELs from the SNP discovery process were considered for the genotyping analysis when present in at least 2 of the "original genotypes" and both strand chromatograms confirmed the polymorphism. Genotyping of the 368 accessions described in Additional file 3 for 80 selected SNPs (including one INDEL) was performed using SNPlex™ (Applied Biosystems) at the Centro Nacional de Genotipado (CeGen [76]). Prior to genotyping, genomic DNAs were re-quantified and normalized at CeGen by means of the PicoGreen technology (Molecular Probes).

## Authors' contributions

DL designed the study, performed sequence and statistical analysis, coordinated the study and drafted the manuscript. JAC performed the PCR primer designs, participated in the design of the study, organized the DNA samples for the genotyping analysis and contributed to the coordination of the study. AI and VR carried out the DNA isolations, PCR amplifications and sample treatments prior to DNA sequencing. JMMZ conceived the study, participated in its design and drafted the manuscript. All authors read and approved the final manuscript.

## Supplementary Material

Additional file 1 **DNA fragments sequenced in this work**. PDF file containing the sequences ID, primers used for their amplification, amplicon size (bp) and associated UniGene or locus identifier. Click here for file

Additional file 2 **SNPs genotyped using SNPlex™**. PDF file containing the SNP IDs, gene diversity, heterozygosity and MAF of the 80 validated SNPs used for the genotyping analysis, together with the MAF observed in the original re-sequenced accessions. Click here for file

Additional file 3 **Grapevine accessions used in this work**. PDF file containing the code, name, main use and repository institution of the grape accessions utilized in the present study. Click here for file

Additional file 4 **Regression plot of MAF in genotyped accession vs. MAF in original accessions**. PDF file displaying the linear regression between the MAF values for the 80 SNPs observed in the sample of ~300 genotyped accessions with the MAF values observed in the original sample of 11 genotypes use in the re-sequencing strategy. Click here for file

Additional file 5 **Global and class specific SNP minor allele frequencies**. PDF file comparing the global minor allele frequency versus those observed in wild, table and wine classes for the 80 validated SNPs tested in the genotyping analysis. Click here for file
