# Supplementary material for: High throughput SNP discovery and genotyping in grapevine (Vitis vinifera L.) by combining a re-sequencing approach and SNPlex technology
Source: BMC Genomics. 2007 Nov 19;8:424. doi: 10.1186/1471-2164-8-424 (PMC2212664; doi:10.1186/1471-2164-8-424)
Supplement: Additional file 1 — DNA fragments sequenced in this work. PDF file containing the sequences ID, primers used for their amplification, amplicon size (bp) and associated UniGene or locus identifier. [file 1471-2164-8-424-S1.pdf]

ID	UniGene or locus identifier	Size (bp)	forward primer	reverse primer
129	Vvi.6889	404	TGTAAAACGACGGCCAGTGGTTTATCATTCACCAGATTGGA	CAGGAAACAGCTATGACCCCTTTTGTGACAGATAACAGGAA
131	Vvi.3977	413	TGTAAAACGACGGCCAGTGGGTGGATAGTTCGTGCAGT	CAGGAAACAGCTATGACCTGGTCCTTGGCATCCTTATT
133	Vvi.3483	211	TGTAAAACGACGGCCAGTAACAAAGCAAACTTCGAAAA	CAGGAAACAGCTATGACCTCTCATCAAGCCAACCTTAC
135	Vvi.3096	469	TGTAAAACGACGGCCAGTTGCCGAGATCATGACAGAAG	CAGGAAACAGCTATGACCTCTCTTTAGCCCCAGCAAGA
189	Vvi.6803	227	TGTAAAACGACGGCCAGTAAAGTCGGTCATCCAAGACA	CAGGAAACAGCTATGACCATCAGCCATTCACCCCTTCT
191	Vvi.552	266	TGTAAAACGACGGCCAGTCAAACAGAAAACAGAAGGAACTTG	CAGGAAACAGCTATGACCGGGCACAAGAGGAAATGAGA
195	Vvi.3458	230	TGTAAAACGACGGCCAGTTTCGCCCTTACACCAGAGG	CAGGAAACAGCTATGACCACATGAAAATAAATACCAAACCACAA
197	Vvi.10605	358	TGTAAAACGACGGCCAGTATGGAGATACAGGGAGGAGT	CAGGAAACAGCTATGACCCAGCTTCCCTACTTATCTGG
205	Vvi.8115	140	TGTAAAACGACGGCCAGTGTAAGGACAAGCATGGTGAG	CAGGAAACAGCTATGACCTCGGTGGTAAAAGTCATAAA
209	Vvi.2857	326	TGTAAAACGACGGCCAGTCACCTCCCACCTCACACAA	CAGGAAACAGCTATGACCTCAGGACAAGGAAATGGAAAA
211	Vvi.8045	770	TGTAAAACGACGGCCAGTTGGGCTTCCTACATTTGCTACT	CAGGAAACAGCTATGACCCGTACAGCTCCTTTGCCTAAAC
215	Vvi.7907	293	TGTAAAACGACGGCCAGTTATCCCCCAAGATGTCAAG	CAGGAAACAGCTATGACCCAAGAAAACAATCAAAATCCA
217	Vvi.4330	384	TGTAAAACGACGGCCAGTAGCACTTCATTTCACTTCTTC	CAGGAAACAGCTATGACCCCATGTGGACTCTTGGAA
219	Vvi.8874	300	TGTAAAACGACGGCCAGTACGACCAGATAAAGGAGACA	CAGGAAACAGCTATGACCCAAGGGTCACAAACAACAC
221	Vvi.2861	354	TGTAAAACGACGGCCAGTCCTCCACATTTTCTGCTGCT	CAGGAAACAGCTATGACCTCTCATTCAGCCTCCTCCAC
227	Vvi.2262	327	TGTAAAACGACGGCCAGTAGAGATACTGTCCGGTGAGA	CAGGAAACAGCTATGACCCTTCATTTGTCCAGGTTTTC
229	Vvi.2364	585	TGTAAAACGACGGCCAGTGAGGTTACTCCAGCCCACATT	CAGGAAACAGCTATGACCCATACCACCCTTCCACCAGT
239	Vvi.1500	491	TGTAAAACGACGGCCAGTGGCTCCATAAGTAGGACTCA	CAGGAAACAGCTATGACCAGCAAGCAAGAGAAGAAGAA
241	Vvi.6717	494	TGTAAAACGACGGCCAGTTGCTTCTTCTCTTCTTCACC	CAGGAAACAGCTATGACCTGAGGTAGCCCTTCTCACTA
243	Vvi.886	370	TGTAAAACGACGGCCAGTTGAAACCAAAGAAGGAACTG	CAGGAAACAGCTATGACCACAACATCAAACCTGCAAA
247	Vvi.1599	322	TGTAAAACGACGGCCAGTGAGCTCCTTCCTTCATTT	CAGGAAACAGCTATGACCTTCCCCTATTGTGTATTGTG
249	CB009615	382	TGTAAAACGACGGCCAGTTGTGTCCTGTAGTGTCTTGG	CAGGAAACAGCTATGACCTGAGAAAGAGAAGGGATTGA
251	Vvi.10077	454	TGTAAAACGACGGCCAGTCCATTCCACCCACCTACAAC	CAGGAAACAGCTATGACCTCAAACCAAACCCAACTGCT
253	Vvi.7545	500	TGTAAAACGACGGCCAGTCGGCGTCGTTTCAGTTTC	CAGGAAACAGCTATGACCGTAGTTGTCTTGCTCGTCGT
255	Vvi.6635	303	TGTAAAACGACGGCCAGTGCACTTTATGCGACCCCTAC	CAGGAAACAGCTATGACCGAAAAAGCAAGCAGTGGAAA
257	Vvi.13149	446	TGTAAAACGACGGCCAGTTAAGGGAGGGATACAAAAAG	CAGGAAACAGCTATGACCGGGCTGCTGTTATCTACAA
259	Vvi.1579	828	TGTAAAACGACGGCCAGTTCCCATTTTCACAACAACCA	CAGGAAACAGCTATGACCGCCGAAGCAGAGGATGTG
261	Vvi.12964	638	TGTAAAACGACGGCCAGTAGGATCAATGGGGTTAAGA	CAGGAAACAGCTATGACCATGAAGGTGAAAGAACGAGT
267	Vvi.11345	176	TGTAAAACGACGGCCAGTAATCTCAAACAATGAACTAAGGA	CAGGAAACAGCTATGACCGTGGGTATGTGGAACGTAAG
269	Vvi.5068	400	TGTAAAACGACGGCCAGTGGGAGCCTTATCAAAGACCA	CAGGAAACAGCTATGACCAAAGGATGGAGGAGGAATGAA
271	Vvi.739	1469	TGTAAAACGACGGCCAGTTCAGGTCCATCACTCTGAGG	CAGGAAACAGCTATGACCGCCTTTGTAAGTCGCAAGG
273	Vvi.8909	713	TGTAAAACGACGGCCAGTGATGGTTTCACCCCAAGTGT	CAGGAAACAGCTATGACCCTTGCTGCAGTTGGTCTCTG
275	Vvi.12240	655	TGTAAAACGACGGCCAGTCGGAGTTCACCGTCCATTAT	CAGGAAACAGCTATGACCTTTCTGTTCGTTCGCTCCTT
279	Vvi.2320	362	TGTAAAACGACGGCCAGTAGCTAATCCCCGCAAGATTT	CAGGAAACAGCTATGACCTGCTTCATCTAAATCCCCACT
281	Vvi.260	374	TGTAAAACGACGGCCAGTCCTCCCATTTCACTCAGCTC	CAGGAAACAGCTATGACCGAGTGGGAAGACTCGTGGTT
283	Vvi.441	388	TGTAAAACGACGGCCAGTAATAGACGGGGCACTGTTTG	CAGGAAACAGCTATGACCTGGTTTATCTTTGGGGTTGG
285	Vvi.587	391	TGTAAAACGACGGCCAGTAGGGGCAAAGGCTAAATGTC	CAGGAAACAGCTATGACCTGGGAGTCAAATTGGAGAGG
289	Vvi.7555	373	TGTAAAACGACGGCCAGTCAAACAGGACAGCTTCTTCCA	CAGGAAACAGCTATGACCCGGCGATTGATTGATTTTG
291	Vvi.898	221	TGTAAAACGACGGCCAGTATCTGGATCCGAGTGATTTC	CAGGAAACAGCTATGACCCAATCAAGAAAATCTTAAACCTC
293	Vvi.6885	194	TGTAAAACGACGGCCAGTTGAACTACCCCATTGAAGA	CAGGAAACAGCTATGACCCTTTATTCATCGCTTTTGCT
297	Vvi.7130	303	TGTAAAACGACGGCCAGTGTAGTCGTGGTCGTTTAGGA	CAGGAAACAGCTATGACCACACACCGTATCCATTCTTC
305	Vvi.2940	259	TGTAAAACGACGGCCAGTGGGCAACCCTGGAGACTACT	CAGGAAACAGCTATGACCACGACCTGCTGATGTGTCTG
311	Vvi.5548	373	TGTAAAACGACGGCCAGTAGCCATTTACCTTCCTTCTT	CAGGAAACAGCTATGACCGGTTCGAGAGCGATACAAT
317	Vvi.6697	296	TGTAAAACGACGGCCAGTACCTTATCCTTACCCACCAC	CAGGAAACAGCTATGACCTACGCTATCAAATCCTGAGC
325	Vvi.7482	228	TGTAAAACGACGGCCAGTACACAAACCAACCAAGAAAG	CAGGAAACAGCTATGACCAAAAAGATGCCTCCTCCA
329	Vvi.7885	323	TGTAAAACGACGGCCAGTCAAAGCCTCCAACCACTGAC	CAGGAAACAGCTATGACCGCAAAAATCAATGCCTTCAAA
331	Vvi.7941	443	TGTAAAACGACGGCCAGTGGGATTGGTTTTTGTTTGTA	CAGGAAACAGCTATGACCAGTTTTGATTTGGATTGCTT
337	Vvi.14385	425	TGTAAAACGACGGCCAGTAAGCCAGACGCTTATACCTCTC	CAGGAAACAGCTATGACCCCCTCTTCTTCCTCTTCTCCA
341	Vvi.1552	367	TGTAAAACGACGGCCAGTCCCACATCACCATCAACATT	CAGGAAACAGCTATGACCGCCAATAAGAGGGAACCAGA
345	Vvi.7060	590	TGTAAAACGACGGCCAGTCAATGAATCTGGGATGAAGT	CAGGAAACAGCTATGACCTCCTGTTTTTCTTGATGGTG
347	Vvi.3139	312	TGTAAAACGACGGCCAGTATCCTCACCATCTCTAAGCA	CAGGAAACAGCTATGACCCATTCCATTTCCTGTATTCC
351	Vvi.4518	372	TGTAAAACGACGGCCAGTAGAGAGCCTTCAAGCAAATG	CAGGAAACAGCTATGACCCGACTTTCTCCCATCAGCAG
355	Vvi.8874	437	TGTAAAACGACGGCCAGTGTTCATCACCCTCGTCTTTA	CAGGAAACAGCTATGACCACGCTCTACTCCACCACTC
357	Vvi.8065	405	TGTAAAACGACGGCCAGTAGGAGAGGAGGCAAAGAAGG	CAGGAAACAGCTATGACCTGAACACTGCAACCAATC
363	Vvi.1275	399	TGTAAAACGACGGCCAGTTCACAGGCCACAAATCCATA	CAGGAAACAGCTATGACCTCAGGGTAGGCGGTATTCTC
365	Vvi.1283	311	TGTAAAACGACGGCCAGTTTGAAGCAGTTCTATGCGAAA	CAGGAAACAGCTATGACCGCACCATTCCACACATCTTG
369	Vvi.1745	398	TGTAAAACGACGGCCAGTCAGAACCAAATCTCGCACCT	CAGGAAACAGCTATGACCCTCTGGAAGAAGGGTTTGTCC
377	Vvi.7795	361	TGTAAAACGACGGCCAGTTAGGGCAACCACTTCCATTC	CAGGAAACAGCTATGACCGCACAAGGGAGAAATACTGG
381	Vvi.1025	119	TGTAAAACGACGGCCAGTCCAGGCAAAGGAAAGTGAAA	CAGGAAACAGCTATGACCTCAAGGGAGCATACAAAAAGG
385	Vvi.1124	374	TGTAAAACGACGGCCAGTACTGTAGCAAGAGGGACCA	CAGGAAACAGCTATGACCAAAGGATTGGGAGGTATTGT
391	Vvi.7339	212	TGTAAAACGACGGCCAGTCGATTCCCTCTCCAACTTCC	CAGGAAACAGCTATGACCTTCCGCACAACAAGGACTG
397	Vvi.6638	358	TGTAAAACGACGGCCAGTCTTCACCGAACTTGACTCC	CAGGAAACAGCTATGACCCTCTCCTCCCCATCATTT
401	Vvi.6643	542	TGTAAAACGACGGCCAGTTGTGGCAGATGAGATGCTG	CAGGAAACAGCTATGACCATCGTTGTTGCGGTTCTTCT
403	Vvi.251	304	TGTAAAACGACGGCCAGTAACGACACCTTCACGCTCTT	CAGGAAACAGCTATGACCTCCAATCTTCCACATCCTCA
405	Vvi.6924	383	TGTAAAACGACGGCCAGTGAATGTCCACCAGACCCATC	CAGGAAACAGCTATGACCACACAGTTTACAGAGCAACAGG
413	Vvi.7386	230	TGTAAAACGACGGCCAGTGCTCTCTTTGTTGCCGTCTC	CAGGAAACAGCTATGACCCCAAATGATACTGCTGGGTTC
415	Vvi.1621	346	TGTAAAACGACGGCCAGTCCTTGTTCATTCCAGCAG	CAGGAAACAGCTATGACCAAGGAGACGATGAGATTGC
421	Vvi.6670	378	TGTAAAACGACGGCCAGTGCCCTTGTCCTCCCAACT	CAGGAAACAGCTATGACCTACTCCCAAATCCGCTCATC
425	Vvi.1402	587	TGTAAAACGACGGCCAGTGTATGCTGCTCCTGCCTGTT	CAGGAAACAGCTATGACCCTGCCGCTTCCTTCATTTC
427	Vvi.8056	702	TGTAAAACGACGGCCAGTTGGACAGAGGATAACATTCC	CAGGAAACAGCTATGACCCCTCACACTCATCAATCTCC
429	Vvi.2290	611	TGTAAAACGACGGCCAGTATAGCAAGCAACCCTCATCG	CAGGAAACAGCTATGACCATCAGCATCTTTGGGTCGTC
437	Vvi.2378	326	TGTAAAACGACGGCCAGTCGCAATGAGACAGAGTGAGA	CAGGAAACAGCTATGACCTTCCAGTTTTCCAGCAGAGG
445	Vvi.7534	439	TGTAAAACGACGGCCAGTCTCACCAGAGAGCAGTACGTGT	CAGGAAACAGCTATGACCCGGCATCCTTCTTCTCATTC
447	Vvi.157	329	TGTAAAACGACGGCCAGTTTGTTCCATTCATTCATTCA	CAGGAAACAGCTATGACCCCAAGGGTCTGTTACCTCTA
451	Vvi.6895	373	TGTAAAACGACGGCCAGTAATCCAGAGAGAGCATTTCC	CAGGAAACAGCTATGACCCACTTATCTTACCGCCATTC
453	Vvi.9104	396	TGTAAAACGACGGCCAGTAACCAAAGAACACACTTGGA	CAGGAAACAGCTATGACCGAACAAGCTCTTACATGGTC
455	Vvi.7590	316	TGTAAAACGACGGCCAGTGGGAACTACTCGGCATTTGT	CAGGAAACAGCTATGACCATGGGGATAAGAACGGGTTG
459	Vvi.7313	422	TGTAAAACGACGGCCAGTAAACAAGCAGTAAGGTTCACA	CAGGAAACAGCTATGACCGGAAATACTCAAAGCCAGTG
463	Vvi.7215	429	TGTAAAACGACGGCCAGTATTGTCTCACTGCTGCGTAT	CAGGAAACAGCTATGACCTCTTGAGTCTGCTTCCAAAT
467	Vvi.7805	688	TGTAAAACGACGGCCAGTCTATCGTGGAGCACATGGAA	CAGGAAACAGCTATGACCCAAACACAGGCATCACATCC
469	Vvi.12905	344	TGTAAAACGACGGCCAGTCTCATCCACCTTCACATTCT	CAGGAAACAGCTATGACCCACTCTCTCTTGCGACAAAC
471	Vvi.6786	372	TGTAAAACGACGGCCAGTCAACCTCATTCCCAAGATAA	CAGGAAACAGCTATGACCTCAATAGCCTTGAACACTGC
473	Vvi.6622	426	TGTAAAACGACGGCCAGTCCACCCACTCTTCTTCACC	CAGGAAACAGCTATGACCGAGCACCCTATTCCCATTCC
477	Vvi.6934	332	TGTAAAACGACGGCCAGTGTCAATAGTCGAATCCAACAC	CAGGAAACAGCTATGACCGTCAAGCAATCAATCCAAAT
479	Vvi.6846	430	TGTAAAACGACGGCCAGTCCGACTTCTTGCCCTACAAC	CAGGAAACAGCTATGACCTGTTTCCCACCTTCATCTCC
487	Vvi.1510	276	TGTAAAACGACGGCCAGTATATCCCAAAGAACCCTAGC	CAGGAAACAGCTATGACCATCTCCCAAGAGGAGGAC
497	Vvi.7873	448	TGTAAAACGACGGCCAGTAGCTGTGTGTGAATGAGCTTG	CAGGAAACAGCTATGACCCCACCAACCTGTCTTCTGCT
503	Vvi.2656	429	TGTAAAACGACGGCCAGTACACAACTGGGACGAGGAAA	CAGGAAACAGCTATGACCGCTGAGGTGGGCTTTCTGTA
517	Vvi.6799	381	TGTAAAACGACGGCCAGTGGAGAACAACAATGGCAACA	CAGGAAACAGCTATGACCGGCACTCCTCGTCCTCAG
519	Vvi.6501	383	TGTAAAACGACGGCCAGTCAGCGAATCCATCAACACAC	CAGGAAACAGCTATGACCGTTTCATCAAGGCCAAGTGC
523	Vvi.7561	395	TGTAAAACGACGGCCAGTAAAGTCAGCATGGGAGTAAA	CAGGAAACAGCTATGACCGTTGGTGTCCTCTTATCTGG
527	Vvi.7374	352	TGTAAAACGACGGCCAGTATCGTTGATGAGGAGGTTGTG	CAGGAAACAGCTATGACCTCTCTGTCCAACTGCCCTCT
533	Vvi.1828	255	TGTAAAACGACGGCCAGTGGACATCAAGCAACCAAACA	CAGGAAACAGCTATGACCCGCAAGAAAGAGGCTAAGGA
537	Vvi.6790	505	TGTAAAACGACGGCCAGTGCCGTAGGAGAAGTTGATGC	CAGGAAACAGCTATGACCGATGCTCGCGTAAGAGACCT
543	Vvi.8117	459	TGTAAAACGACGGCCAGTGACAAGAAGACCCGCCATTA	CAGGAAACAGCTATGACCCATCACGGCCTATGTTCAAA
551	Vvi.5062	535	TGTAAAACGACGGCCAGTCATCTTCTTCCCGTCAGTGG	CAGGAAACAGCTATGACCACAAGTCGGAGTGGGTGAAG
553	Vvi.2453	361	TGTAAAACGACGGCCAGTCTTCCCACTGAGGCTACGA	CAGGAAACAGCTATGACCCCACTAAGCACACCCACAAA
555	Vvi.1163	453	TGTAAAACGACGGCCAGTTGCCTCCCAGTTCTTCTACC	CAGGAAACAGCTATGACCCATTACCAACCTGCCGAAGT
557	Vvi.7811	405	TGTAAAACGACGGCCAGTGCCTGAACAAAGAAGCCAAA	CAGGAAACAGCTATGACCGCTTGAGGTCATCCACTGC
559	Vvi.1222	254	TGTAAAACGACGGCCAGTTTACCGCCCTTGTCCTTGTA	CAGGAAACAGCTATGACCGCGTATTGATCATCCCTTGG
561	Vvi.12074	279	TGTAAAACGACGGCCAGTAGGAGGCCTATGCATCAGAA	CAGGAAACAGCTATGACCGGAGCACCATTAAAGGCAAA
565	Vvi.3208	309	TGTAAAACGACGGCCAGTGCATATAGCACAGCAAAGC	CAGGAAACAGCTATGACCGAGAACTTGGAGAAGAAGCA
567	Vvi.7830	459	TGTAAAACGACGGCCAGTTCTCTCTCCGTTCTTCTTTG	CAGGAAACAGCTATGACCGGTTTGTGTACTTCTTCCTT
569	Vvi.7646	375	TGTAAAACGACGGCCAGTAATCCAAACTCCCCACCAC	CAGGAAACAGCTATGACCCTTCTCGGCTGTGTCCATCT
571	Vvi.11189	371	TGTAAAACGACGGCCAGTATCAACATACACAGATCGACAA	CAGGAAACAGCTATGACCCCATCATTGGGAAACTCA
573	Vvi.4522	302	TGTAAAACGACGGCCAGTTCCTCCCTACAAATGAGC	CAGGAAACAGCTATGACCCAAAATCAAAGAAGCCTACAG
575	Vvi.6640	270	TGTAAAACGACGGCCAGTCCTTGGCATTGTATGTTTGA	CAGGAAACAGCTATGACCTGGTGAAGAACCTGTGGTCA
579	Vvi.3568	300	TGTAAAACGACGGCCAGTTTCTGTGTGGGAAGTTGCTG	CAGGAAACAGCTATGACCGGATTGGAGTAGCCATCTGC
581	Vvi.7828	350	TGTAAAACGACGGCCAGTCAAACCCATAAGCACTCCAAA	CAGGAAACAGCTATGACCCACTTTGGTCAGCTCCACCT
591	Vvi.8089	391	TGTAAAACGACGGCCAGTCCGGTTAAGTTTGATATGACTG	CAGGAAACAGCTATGACCAAGAACCTTGCCACGAAG
593	Vvi.6936	343	TGTAAAACGACGGCCAGTTGGATGTGCCTAAGAGATG	CAGGAAACAGCTATGACCATGATGCTCCTAAAGATGCT
595	Vvi.12406	364	TGTAAAACGACGGCCAGTGCTTCCTACGCTCTCCTTCA	CAGGAAACAGCTATGACCTGCCATTCATGCTTCTTTACA
599	Vvi.15592	511	TGTAAAACGACGGCCAGTGCCACCAAGAAGAAAGGAGA	CAGGAAACAGCTATGACCCAACACCAATGACACCTCCA
603	Vvi.8069	422	TGTAAAACGACGGCCAGTTCTTTCGTTCTATCCAGTAATGT	CAGGAAACAGCTATGACCCCTTCTTCCATTTATCCACA
605	Vvi.7461	319	TGTAAAACGACGGCCAGTCTTCCTTGGGCTATGATTC	CAGGAAACAGCTATGACCCCTTCATGTGCTCTCTCCT
613	Vvi.7462	390	TGTAAAACGACGGCCAGTGCTGACACAACTCCACGAAC	CAGGAAACAGCTATGACCGCCTGAGCCTACCTTCTCAA
621	Vvi.3373	410	TGTAAAACGACGGCCAGTAGGAAAACATCCCACAATAA	CAGGAAACAGCTATGACCCCTCATCGTCTCTGTGATTT
625	Vvi.6997	350	TGTAAAACGACGGCCAGTCTTGGACCTCAGCCTTGTTC	CAGGAAACAGCTATGACCTCATCGTCTTCACTCTCCTTCA
631	CB002658	395	TGTAAAACGACGGCCAGTGATTACAAGGCTCAGATTCC	CAGGAAACAGCTATGACCATGAAGAGAAAGTGTGACCTG
633	Vvi.7378	255	TGTAAAACGACGGCCAGTCCATCACCTTCCAAATTAAA	CAGGAAACAGCTATGACCGAGATAACTTCGGGCCTTAT
635	Vvi.738	444	TGTAAAACGACGGCCAGTCTCTTCTCCGTCTTCTACCA	CAGGAAACAGCTATGACCAAACAGAGCCAAAACAAAAC
643	Vvi.10410	460	TGTAAAACGACGGCCAGTTAAATATGCCCATGTCCTTT	CAGGAAACAGCTATGACCAAGTGGTTCAAAGATTACGAG
649	Vvi.8076	755	TGTAAAACGACGGCCAGTCTGGATGCTCTGCTTGTGA	CAGGAAACAGCTATGACCTTCTCTTATGCCGCTTCTGC
651	Vvi.6647	658	TGTAAAACGACGGCCAGTTCATAACCAGCAAGCACACC	CAGGAAACAGCTATGACCCAATGATGCTAAGGCTGCTAAA
653	Vvi.316	406	TGTAAAACGACGGCCAGTCAATGAAAGCAACCTACACA	CAGGAAACAGCTATGACCATTCTCCATTTGACACCAAC
655	Vvi.6780	366	TGTAAAACGACGGCCAGTTCGTCAAGAACAGGTCCACA	CAGGAAACAGCTATGACCCAAAGCCGTCAAGTACATCG
659	CB970655	733	TGTAAAACGACGGCCAGTCTGAGACAGGGAGTGGAATA	CAGGAAACAGCTATGACCGCAGCATACAACAAGAAACA
663	Vvi.7589	681	TGTAAAACGACGGCCAGTGGTCGGTGAGGATGAGAAGA	CAGGAAACAGCTATGACCTTGGCGAGATAAGAGCAGTC

ID	UniGene or locus identifier	Size (bp)	forward primer	reverse primer
677	Vvi.3315	611	TGTAAAACGACGGCCAGTCCTCTACAAGATTGCGGATA	CAGGAAACAGCTATGACCAGATGTTCAGCCAGTAAAGC
683	Vvi.7884	302	TGTAAAACGACGGCCAGTCCAGTCTTGAACCGCTCCT	CAGGAAACAGCTATGACCGTGATTGTCCGCTCTTTCG
689	Vvi.5832	338	TGTAAAACGACGGCCAGTGGAAGCTCGTAGCATTGG	CAGGAAACAGCTATGACCTATTATGGCTTGGCCCTTT
691	Vvi.4484	446	TGTAAAACGACGGCCAGTCTCGCAAAGAACATAAGAGCA	CAGGAAACAGCTATGACCCAGAGCAGAAGGGCACATTA
697	Vvi.1323	398	TGTAAAACGACGGCCAGTAAGACATCGCCAAAGAGACC	CAGGAAACAGCTATGACCGAAGCAAACCGTAGCAATCC
699	Vvi.7621	423	TGTAAAACGACGGCCAGTGCAATGGTGTTAAGAACCTC	CAGGAAACAGCTATGACCACTCACTCGCTCACTCACTC
701	Vvi.8086	475	TGTAAAACGACGGCCAGTTGTAGTTGTGAGCGAGTTTG	CAGGAAACAGCTATGACCCTTTAGTTTGCCTTTCAGGA
705	Vvi.7405	290	TGTAAAACGACGGCCAGTCCTAGTGACCCATACCAGAG	CAGGAAACAGCTATGACCACCAAACCTCTTTCTCATCC
709	Vvi.4581	476	TGTAAAACGACGGCCAGTTACAGCGTCAACCTCACTGC	CAGGAAACAGCTATGACCCAGTCCCAGTCTCCTTCTTCC
713	Vvi.7970	758	TGTAAAACGACGGCCAGTGGAGGAGTTTAGGAAGCAG	CAGGAAACAGCTATGACCCTCCCTTGTTGACATCCTT
715	Vvi.1493	331	TGTAAAACGACGGCCAGTTCACCTTGAAAGCCCTAACC	CAGGAAACAGCTATGACCACTATGCCGCTTGTTTGCTT
729	Vvi.8105	387	TGTAAAACGACGGCCAGTTATCGCAAGCCCTAGATATG	CAGGAAACAGCTATGACCGGAGGTAAGAACAGGAAGAGA
733	Vvi.1569	252	TGTAAAACGACGGCCAGTCCAACCAAATGGAATGGATTA	CAGGAAACAGCTATGACCGGAACCACAACCCTCCAGT
741	Vvi.1527	309	TGTAAAACGACGGCCAGTTCTTGTTCTTGTTGCATCTC	CAGGAAACAGCTATGACCTCACAGAAATTGAAACTGAAG
745	Vvi.6724	377	TGTAAAACGACGGCCAGTGGAGTTGGAGGTTTGTGTGG	CAGGAAACAGCTATGACCGTTGCTTGGAGTGGTGGTTC
751	Vvi.133	419	TGTAAAACGACGGCCAGTACATTCCAGGCACGACAGAT	CAGGAAACAGCTATGACCGATCATTCAAGTTACAGGTG
753	AF378127	1686	TGTAAAACGACGGCCAGTGTCCAGTCCCACAGCCTC	CAGGAAACAGCTATGACCGTTGACCTCTGGGACTCG
759	Vvi.824	934	TGTAAAACGACGGCCAGTGTAGACCCAACACCAGCAC	CAGGAAACAGCTATGACCGTCTTAATCGCTCGTTCTC
765	CV093472	1476	TGTAAAACGACGGCCAGTGGATATACTTGGAAGGTACACTATGC	CAGGAAACAGCTATGACCTTGTTGTTCTCTTCCATCAG
775	AM434138	1686	TGTAAAACGACGGCCAGTTCACTTGGAGGGAGAAACT	CAGGAAACAGCTATGACCTCCAGCAAAATACAATTCCT
781	Vvi.2405	1089	TGTAAAACGACGGCCAGTGCAGGTCTTTGCCTTGTT	CAGGAAACAGCTATGACCCATTACAGGAGAAAAGCACA
787	Vvi.11892	1117	TGTAAAACGACGGCCAGTAAGCAGGATGCAGAAAGC	CAGGAAACAGCTATGACCACTTGGTTGTGCTGAGAGTT
799	Vvi.129	537	TGTAAAACGACGGCCAGTCTCATCTGCCTATGTGGT	CAGGAAACAGCTATGACCTAACTAATGGTGGTGCAGTG
801	Vvi.13738	490	TGTAAAACGACGGCCAGTATCTACTTCAACTGCTCTTTGC	CAGGAAACAGCTATGACCTATTGGTTCAAGCGTAAGGT
811	Vvi.128	465	TGTAAAACGACGGCCAGTAGACTTGGATCACCGTTCAA	CAGGAAACAGCTATGACCAAGCAGGAGGAGTGGACAA
815	Vvi.8863	418	TGTAAAACGACGGCCAGTAAGCCTCAAATTCAAAGTGT	CAGGAAACAGCTATGACCGCGTTTCTTTCATTCTCTCTCG
817	Vvi.1581	437	TGTAAAACGACGGCCAGTCGTATCAATGTAGAGCAGCA	CAGGAAACAGCTATGACCGTTCAGCAAGAAGGAAGTTG
819	Vvi.1591	372	TGTAAAACGACGGCCAGTGCAAAGGTCCAGAGGGAGTT	CAGGAAACAGCTATGACCTGATGAAAGGGAAAGGAGTGA
825	Vvi.1560	359	TGTAAAACGACGGCCAGTTCAAGGTTCAGGTGGAGGAC	CAGGAAACAGCTATGACCCTATGTGGAAGCCGATGACC
827	Vvi.14739	247	TGTAAAACGACGGCCAGTCATGTGGCCTTGCTTGGT	CAGGAAACAGCTATGACCAATAGCGAACAACCTTTGC
829	Vvi.1206	373	TGTAAAACGACGGCCAGTAGACGGAGGAAGAGAAGAAG	CAGGAAACAGCTATGACCCTGAACCCAGAAGTCAAGAG
835	Vvi.10423	275	TGTAAAACGACGGCCAGTACTTTCCCATTCATGATGTC	CAGGAAACAGCTATGACCGCTGAATTTTGCTTTCATGG
837	Vvi.1551	340	TGTAAAACGACGGCCAGTACACACCCACAATACCACAT	CAGGAAACAGCTATGACCCTACGCCTTCATCATCGAC
841	Vvi.8949	443	TGTAAAACGACGGCCAGTCATCACCCAAATTCCCAAAT	CAGGAAACAGCTATGACCCGAGCAGAGAACGAGTGAGA
843	Vvi.398	417	TGTAAAACGACGGCCAGTAACTTCCTCTGCGCCATAA	CAGGAAACAGCTATGACCGCTGACCACGATTGGACTTC
845	Vvi.8153	876	TGTAAAACGACGGCCAGTTTCACACAGCAGGATCTTC	CAGGAAACAGCTATGACCCCATCTCCAACAGCAAAGGT
851	Vvi.1191	348	TGTAAAACGACGGCCAGTCCACGTTATTGCTGCTTC	CAGGAAACAGCTATGACCACTTGTCATGGAAGGTTACAA
853	Vvi.8888	346	TGTAAAACGACGGCCAGTGCAAGGACATGGCTTATATT	CAGGAAACAGCTATGACCAACTAACACCCTGAATGGAC
855	Vvi.7925	202	TGTAAAACGACGGCCAGTACCACCTGTCACCACCAAC	CAGGAAACAGCTATGACCCTCCGCATCAGAAGTCATCA
857	Vvi.6159	436	TGTAAAACGACGGCCAGTTGAGGAGACCATAGACCATC	CAGGAAACAGCTATGACCAAGGCGACTCAAGAAATACA
859	Vvi.7639	398	TGTAAAACGACGGCCAGTCTCACACTGCCCTCACTG	CAGGAAACAGCTATGACCCTCAACGCTTCAGACACTC
865	Vvi.2316	557	TGTAAAACGACGGCCAGTGCTGTGCTATGTTGTTATCG	CAGGAAACAGCTATGACCGCTTGTATGCTGGTCTTCTT
867	Vvi.9994	778	TGTAAAACGACGGCCAGTGAAGTAGTGGGTGAGGTTGA	CAGGAAACAGCTATGACCTCTGGGCTAATGATGAAAGT
871	Vvi.6728	376	TGTAAAACGACGGCCAGTCACACTGGTTGCAGTCACAT	CAGGAAACAGCTATGACCGTTCACCGGCTTCTAATCAC
873	Vvi.9410	435	TGTAAAACGACGGCCAGTCCGAAATAGAGGCAGCAAGA	CAGGAAACAGCTATGACCATGTTTCCACCCACCAAGG
875	Vvi.1711	359	TGTAAAACGACGGCCAGTCGACCGTTAGGAGATGCTGT	CAGGAAACAGCTATGACCACGTCTGGTTCGATGTAGCC
877	Vvi.7410	427	TGTAAAACGACGGCCAGTGGAGTCACATGGCAACAGAA	CAGGAAACAGCTATGACCTGGATTGGTAGATCGCTACG
879	Vvi.9151	378	TGTAAAACGACGGCCAGTGCAAATCCACCGATACCAAC	CAGGAAACAGCTATGACCCGCCAGCTATGAACACCTTC
881	Vvi.7982	487	TGTAAAACGACGGCCAGTCATGGCAACCAAGTACATAA	CAGGAAACAGCTATGACCAACTGTGTGCAAATCAAAGG
883	Vvi.9204	691	TGTAAAACGACGGCCAGTGCTCCTTTTCGCTCATTCTT	CAGGAAACAGCTATGACCACATTCCACTGCTCGCTCTT
887	Vvi.2222	323	TGTAAAACGACGGCCAGTTGCCTTGTGTGTGCTTTCTC	CAGGAAACAGCTATGACCGAATCATCTCAGCAGCCTCA
891	Vvi.5254	378	TGTAAAACGACGGCCAGTCTCAGCAGTCACGCAAGATT	CAGGAAACAGCTATGACCATTTCATCAGCAGCCACCA
893	Vvi.7958	517	TGTAAAACGACGGCCAGTGACAGGCAAGAGCGTTTCAT	CAGGAAACAGCTATGACCGCCTTCAACCGTAGGATTTG
895	Vvi.6840	423	TGTAAAACGACGGCCAGTGCTGCAACAGAAACAGAGCTT	CAGGAAACAGCTATGACCAAAGCACATCTGGCAACCTT
897	Vvi.893	422	TGTAAAACGACGGCCAGTGTCGAAGTGGTTGTGGAT	CAGGAAACAGCTATGACCCGAGTTCTTCACCTTCACGA
907	Vvi.7571	443	TGTAAAACGACGGCCAGTAAACGGAACGGAAGAAGG	CAGGAAACAGCTATGACCATTTACAGTGAGGCAAGGAA
915	Vvi.7389	438	TGTAAAACGACGGCCAGTCACCTGGATCAACACACCAG	CAGGAAACAGCTATGACCATTACCCGCGCTAGATTCG
919	Vvi.4117	470	TGTAAAACGACGGCCAGTGGAAATGGTGGCAAGTAAGG	CAGGAAACAGCTATGACCCCCACTGACCTTGTCGTCTT
923	Vvi.6727	371	TGTAAAACGACGGCCAGTGCTCCGAACAACTACGCTTC	CAGGAAACAGCTATGACCCCATCGTCCAGTCACACATC
925	Vvi.10211	363	TGTAAAACGACGGCCAGTTCTCCTCCTCTCCACCAGAA	CAGGAAACAGCTATGACCCAGAACATCCTGCATAGCATT
929	Vvi.10340	329	TGTAAAACGACGGCCAGTCTCTTTCCCTTCTCTTCTTTGC	CAGGAAACAGCTATGACCGCATCTCCCTCTCTCCAG
933	Vvi.1699	542	TGTAAAACGACGGCCAGTTTGAAGGAGAACAGGGAGGA	CAGGAAACAGCTATGACCAGCGCCTTAACAGCCTTTG
941	Vvi.7707	480	TGTAAAACGACGGCCAGTGCTATTGGACGTGTATGTGA	CAGGAAACAGCTATGACCACAAGGAGCCAAGAACATTA
945	CF215168	355	TGTAAAACGACGGCCAGTGGTTGCCTCGGATTTGATTA	CAGGAAACAGCTATGACCGCCTTGCCACACCTGAAA
947	Vvi.7240	345	TGTAAAACGACGGCCAGTAGGAGGCAGAAGCAGAGA	CAGGAAACAGCTATGACCCCTTTACCATTCATCATAGC
961	Vvi.2190	299	TGTAAAACGACGGCCAGTGCAAGCAGAACTGAAGCA	CAGGAAACAGCTATGACCGCCTCCAATTACAATATCCA
967	Vvi.3288	341	TGTAAAACGACGGCCAGTGAACGATGACGAAAGCACAA	CAGGAAACAGCTATGACCGCATTTCCCAGTCAAACGAG
969	Vvi.6678	175	TGTAAAACGACGGCCAGTATCATCGTCACATTCCAC	CAGGAAACAGCTATGACCCCACTCTACAACATCAAGG
973	Vvi.10243	388	TGTAAAACGACGGCCAGTCATCATCTACACAGCAGGAACC	CAGGAAACAGCTATGACCAAAGCGTCATCGGAAAGTTCT
977	Vvi.7332	420	TGTAAAACGACGGCCAGTAGGAGAAGCACCACAAGCAT	CAGGAAACAGCTATGACCGACCCAGGAAACAAGACACAA
987	Vvi.10197	361	TGTAAAACGACGGCCAGTATGAATGCCAAGCAGGAGAT	CAGGAAACAGCTATGACCTTAACCACACCAGCACCAAC
1001	Vvi.7740	434	TGTAAAACGACGGCCAGTGGAATGCAAGGACAGGAAC	CAGGAAACAGCTATGACCCACCACCAGAGTCATCCAA
1003	Vvi.1292	500	TGTAAAACGACGGCCAGTCCAAGTTTCCTGTGGCATT	CAGGAAACAGCTATGACCAGAGGTTGTCTGGAGGCTGA
1007	Vvi.11667	585	TGTAAAACGACGGCCAGTGAAACAGCACAAGATAGAAACC	CAGGAAACAGCTATGACCCGGAGCTTGATACAGAAGG
1009	Vvi.12361	421	TGTAAAACGACGGCCAGTCAGTCGCCAGGTTATGGATT	CAGGAAACAGCTATGACCTGCGGAGTACAGTTGAAGGA
1011	Vvi.3190	538	TGTAAAACGACGGCCAGTTCTGGTGCTATCCGTGTTC	CAGGAAACAGCTATGACCCAGCTCTTCCAACTCCTCA
1015	Vvi.8123	333	TGTAAAACGACGGCCAGTTCCAAAACGTAGCAGCAAGA	CAGGAAACAGCTATGACCCCAGGATGGTAACGAGGTGT
1017	Vvi.668	304	TGTAAAACGACGGCCAGTTGCCAAGACAGATGCACATAG	CAGGAAACAGCTATGACCAGGCAGTGGTGCTAAAGAGG
1021	Vvi.7575	218	TGTAAAACGACGGCCAGTAAAACTGAAACGATGAGTGC	CAGGAAACAGCTATGACCGCGGAGATTGAGAAGAGGA
1023	Vvi.41	487	TGTAAAACGACGGCCAGTCCGCACTCAAACAACAGGTA	CAGGAAACAGCTATGACCTACTCTCACCAGCTCGCACA
1025	Vvi.7021	444	TGTAAAACGACGGCCAGTAATCAACGCAAACAAAGACG	CAGGAAACAGCTATGACCTGCTCAACTCTCTGCGAAA
1027	Vvi.5643	628	TGTAAAACGACGGCCAGTAGGCACCAAACCACAACTTC	CAGGAAACAGCTATGACCATCCAAGAGGCTGACCAGAG
1029	Vvi.8007	316	TGTAAAACGACGGCCAGTATGGCTGAGAAGATGGTGT	CAGGAAACAGCTATGACCGAATGTTGATATTGAGCTTGC
1031	CF516996	441	TGTAAAACGACGGCCAGTCTTCTTCTTCTTCTCAATCAACC	CAGGAAACAGCTATGACCCCAAGGCACTTCTCCACTT
1071	Vvi.461	283	TGTAAAACGACGGCCAGTGCCGTTCTTCTTCAGAGC	CAGGAAACAGCTATGACCTCAGGACTGTTTGATCGAG
1109	Vvi.6764	354	TGTAAAACGACGGCCAGTCAGAAGTCATTGGAGGTTGC	CAGGAAACAGCTATGACCGGAAGCAGCAAGACAGATCC
1119	Vvi.10	357	TGTAAAACGACGGCCAGTTTAGGCCAGAAGGATGGCTA	CAGGAAACAGCTATGACCACAAAGACCCTTGGGACCAT
1127	Vvi.85	435	TGTAAAACGACGGCCAGTCAAGCCTATTGCGTCTGTCA	CAGGAAACAGCTATGACCGTCCACTGCCACCAGAAT
1137	Vvi.129	609	TGTAAAACGACGGCCAGTGAGCAAAACAACGCATTAGC	CAGGAAACAGCTATGACCTGGATTTATCTGGGGCATT
1151	Vvi.1307	551	TGTAAAACGACGGCCAGTCGCTCGAATTAGTTGTAAGG	CAGGAAACAGCTATGACCGATGCTTTCTGCTTGGATG
1157	Vvi.2154	439	TGTAAAACGACGGCCAGTGGTAGAGATGAAGATGAGAGC	CAGGAAACAGCTATGACCTACTCAAGCCTGTGGAATG
1161	Vvi.3370	373	TGTAAAACGACGGCCAGTCGTGCAGGCCAGAGTGAG	CAGGAAACAGCTATGACCCCGATTCCACCTCTAGTGAAG
1171	Vvi.7047	355	TGTAAAACGACGGCCAGTCGAGACAGATTGGTTGAATGC	CAGGAAACAGCTATGACCCTTTCCCTCGATTTCTTTG
1175	Vvi.7185	347	TGTAAAACGACGGCCAGTGGATGGTGAAGAGAGGTGA	CAGGAAACAGCTATGACCGGTTCCCAGTGCTAGTAAA
1191	Vvi.11961	201	TGTAAAACGACGGCCAGTACACACTCAGCTCCGGTAGG	CAGGAAACAGCTATGACCGGCAACAAGGTCAGCAACA
1201	Vvi.12037	360	TGTAAAACGACGGCCAGTTCTCTTTGGATTGGCGTAGG	CAGGAAACAGCTATGACCGTGTAACGCACTCAGCAAGC
1211	Vvi.12138	202	TGTAAAACGACGGCCAGTCACAAGATTTGAAGTGACCA	CAGGAAACAGCTATGACCGAGTTGGTTTCGTGAGAATTG
1213	Vvi.12158	609	TGTAAAACGACGGCCAGTTTCACCATCAGGCACAAAGG	CAGGAAACAGCTATGACCGGCGGTGGAAATAGAGTTG
1215	Vvi.12195	201	TGTAAAACGACGGCCAGTGTGTTAGAATGCCTCTTGG	CAGGAAACAGCTATGACCTGTCATGGAGTGGTTTAAGA
1217	Vvi.12233	220	TGTAAAACGACGGCCAGTTGCTGATGAAGGAAGTTTCG	CAGGAAACAGCTATGACCCCGTATTCTGGACAGGAGGA
1229	Vvi.12341	320	TGTAAAACGACGGCCAGTCCTCTCAGCTCTACCACAAGC	CAGGAAACAGCTATGACCCTTCACATCCACAACCTC
1	AF450278	1748	TGTAAAACGACGGCCAGTTCACTCCTCACTCCTCTCTG	CAGGAAACAGCTATGACCCATTGGAAGCCTCCTCATCA
