# Supplementary material for: High throughput SNP discovery and genotyping in grapevine (Vitis vinifera L.) by combining a re-sequencing approach and SNPlex technology
Source: BMC Genomics. 2007 Nov 19;8:424. doi: 10.1186/1471-2164-8-424 (PMC2212664; doi:10.1186/1471-2164-8-424)
Supplement: Additional file 2 — SNPs genotyped using SNPlex™. PDF file containing the SNP IDs, gene diversity, heterozygosity and MAF of the 80 validated SNPs used for the genotyping analysis, together with the MAF observed in the original re-sequenced accessions. [file 1471-2164-8-424-S2.pdf]

SNP ID	Genotyped accessions			Original accessions
	Gene diversity	Heterozygosity	MAF	MAF
SNP1_351	0.30	0.13	0.17	0.25
SNP129_237	0.45	0.32	0.32	0.45
SNP135_316	0.37	0.30	0.26	0.40
SNP189_131	0.32	0.34	0.25	0.15
SNP191_100	0.31	0.23	0.17	0.11
SNP197_82	0.47	0.51	0.36	0.41
SNP209_255	0.30	0.26	0.18	0.25
SNP217_190	0.27	0.26	0.14	0.14
SNP227_191	0.42	0.33	0.30	0.32
SNP229_112	0.48	0.48	0.36	0.45
SNP241_201	0.28	0.27	0.21	0.25
SNP249_125	0.15	0.16	0.08	0.09
SNP255_265	0.46	0.49	0.37	0.40
SNP259_199	0.48	0.44	0.40	0.45
SNP269_308	0.50	0.44	0.50	0.40
SNP273_298	0.27	0.27	0.16	0.14
SNP273_361	0.10	0.11	0.06	0.05
SNP273_469	0.07	0.07	0.03	0.14
SNP281_64	0.44	0.38	0.33	0.36
SNP283_32	0.21	0.18	0.14	0.10
SNP289_84	0.42	0.39	0.33	0.25
SNP293_20	0.12	0.12	0.07	0.15
SNP311_198	0.49	0.34	0.42	0.32
SNP317_155	0.34	0.31	0.24	0.45
SNP325_65	0.28	0.26	0.20	0.19
SNP341_196	0.45	0.43	0.35	0.40
SNP345_421	0.09	0.09	0.04	0.07
SNP351_85	0.40	0.38	0.30	0.50
SNP355_154	0.49	0.42	0.44	0.33
SNP357_371	0.49	0.48	0.45	0.25
SNP377_251	0.47	0.50	0.37	0.45
SNP391_170	0.24	0.24	0.12	0.08
SNP397_331	0.24	0.20	0.13	0.20
SNP415_209	0.28	0.28	0.16	0.25
SNP421_234	0.25	0.17	0.13	0.14
SNP425_205	0.12	0.09	0.06	0.09
SNP437_129	0.31	0.25	0.19	0.40
SNP447_244	0.47	0.44	0.45	0.40
SNP451_287	0.45	0.45	0.35	0.36
SNP453_375	0.49	0.48	0.44	0.50
SNP457_192	0.10	0.09	0.04	0.10
SNP459_140	0.36	0.36	0.22	0.33
SNP463_296	0.05	0.05	0.02	0.15
SNP477_239	0.29	0.21	0.16	0.21
SNP497_281	0.12	0.12	0.07	0.15
SNP517_224	0.48	0.34	0.39	0.45
SNP533_161	0.02	0.02	0.01	0.07
SNP543_268	0.19	0.20	0.10	0.23
SNP551_351	0.23	0.23	0.12	0.28
SNP553_98	0.47	0.40	0.39	0.27
SNP555_132	0.47	0.41	0.38	0.38
SNP557_104	0.29	0.23	0.17	0.15
SNP559_110	0.37	0.37	0.23	0.50
SNP561_120	0.07	0.06	0.03	0.07
SNP567_341	0.35	0.41	0.21	0.15
SNP571_227	0.50	0.56	0.48	0.36
SNP575_128	0.26	0.27	0.14	0.25
SNP579_187	0.23	0.22	0.14	0.09
SNP581_114	0.46	0.57	0.39	0.32
SNP591_148	0.45	0.43	0.35	0.27
SNP593_149	0.47	0.47	0.39	0.32
SNP605_120i	0.24	0.21	0.14	0.18
SNP613_315	0.44	0.50	0.33	0.20
SNP625_278	0.13	0.10	0.08	0.09
SNP635_21	0.27	0.22	0.16	0.13
SNP649_567	0.49	0.36	0.40	0.50
SNP651_658	0.03	0.03	0.01	0.14
SNP653_90	0.44	0.31	0.30	0.23
SNP655_93	0.28	0.26	0.16	0.23
SNP659_73	0.48	0.41	0.40	0.32
SNP683_120	0.08	0.07	0.05	0.09
SNP691_139	0.50	0.41	0.49	0.27
SNP697_296	0.26	0.23	0.14	0.14
SNP699_311	0.28	0.24	0.14	0.17
SNP709_258	0.38	0.40	0.25	0.33
SNP715_260	0.10	0.11	0.06	0.20
SNP811_42	0.39	0.43	0.28	0.40
SNP817_209	0.12	0.13	0.07	0.11
SNP819_210	0.49	0.47	0.43	0.45
SNP829_281	0.50	0.44	0.48	0.50
**Mean**	**0.32**	**0.30**	**0.24**	**0.26**
