# Supplementary material for: High throughput SNP discovery and genotyping in grapevine (Vitis vinifera L.) by combining a re-sequencing approach and SNPlex technology
Source: BMC Genomics. 2007 Nov 19;8:424. doi: 10.1186/1471-2164-8-424 (PMC2212664; doi:10.1186/1471-2164-8-424)
Supplement: Additional file 3 — Grapevine accessions used in this work. PDF file containing the code, name, main use and repository institution of the grape accessions utilized in the present study. [file 1471-2164-8-424-S3.pdf]

Code	Name	Main use	Repository
14A22	Clairette Blanche	wine	IMIDRA[1]
14B14	Muscat Ottonel	table	IMIDRA
14B17	Muscat Santa Vallier	table	IMIDRA
14B22	Mondina	wine	IMIDRA
14D20	Segregada 22-52	table	IMIDRA
14E29	Doroni Maceron	table	IMIDRA
14F05	Kalili	table	IMIDRA
14F10	Foster's White	table	IMIDRA
14I07	Red Ohanes	table	IMIDRA
22A19	Morisca	wine	IMIDRA
22A21	Maturana	wine	IMIDRA
22A31	Ribote	wine	IMIDRA
22A32	Verues de Huarte	wine	IMIDRA
22A37	Jeronimo de Tudela	wine	IMIDRA
22A38	Redondal	table	IMIDRA
22A40	Tempranillo	wine	IMIDRA
22A43	Graciano	wine	IMIDRA
22A44	Mazuelo	wine	IMIDRA
22B01	Negra Tardía	table	IMIDRA
22B02	Ragol	table	IMIDRA
22B03	Ciruela Roja	table	IMIDRA
22B04	Dominga	table	IMIDRA
22B05	Molinera	table	IMIDRA
22B10	Palomino de Jerez	wine	IMIDRA
22B12	Palomino 84	wine	IMIDRA
22B18	Gabriela	wine	IMIDRA
22B32	Torrontes	wine	IMIDRA
22B35	Vijiriega Comun	wine	IMIDRA
22B45	Plateado	wine	IMIDRA
22C01	Negra Rayada	wine	IMIDRA
22C05	Imperial	table	IMIDRA
22C08	Jaen Negro	wine	IMIDRA
22C11	Zalema	wine	IMIDRA
22C14	Rocia	wine	IMIDRA
22C15	Pedro Luis	wine	IMIDRA
22C16	Listan Blanca	wine	IMIDRA
22C18	Garrido Macho	wine	IMIDRA
22C22	Del Rey	wine	IMIDRA
22C24	Beba	table	IMIDRA
22C26	Garrido Fino	wine	IMIDRA
22C27	Perruna	wine	IMIDRA
22C43	Cojonata	wine	IMIDRA
22C44	Moscatel Negro	table	IMIDRA
22C45	Moscatel Malaga	table	IMIDRA
22C50	Rey	wine	IMIDRA
22C58	Mollar	wine	IMIDRA
22E05	Quiebratinajas Blanco	wine	IMIDRA
22E08	Cadrete	wine	IMIDRA
22G04	Tetona Blanca	wine	IMIDRA
22G38	Cariñena Blanca	wine	IMIDRA
22H17	Tarragoni	wine	IMIDRA
22H19	Garnacha Peluda	wine	IMIDRA
22H49	Albillo	wine	IMIDRA
22H51	Rojal Blanco	wine	IMIDRA
22I11	Rojal	wine	IMIDRA
22I34	Rojal	wine	IMIDRA
22J50	Tinto Navalcarnero	wine	IMIDRA
22K17	Gualarido	table	IMIDRA
22K38	Albillo Mayor	wine	IMIDRA
22L30	Garnacho	wine	IMIDRA
22L40	Verdejo	wine	IMIDRA
22L43	Salvador	wine	IMIDRA
22M07	Morisca	wine	IMIDRA
22M12	Fernán Piriz	wine	IMIDRA
22M17	Eva	table	IMIDRA
22M35	Albillo Real	wine	IMIDRA
22N21	Treixadura	wine	IMIDRA
22N56	Aledo	table	IMIDRA
22O10	Bobal	wine	IMIDRA
22O11	Monastrell	wine	IMIDRA
22O18	More	table	IMIDRA
22O22	Mondragon	wine	IMIDRA
22O23	Castellano	wine	IMIDRA
22O27	Messeguera	wine	IMIDRA
22O44	Grumiere Blanco	wine	IMIDRA
22O46	Gateta	table	IMIDRA
22O52	Morenillo	wine	IMIDRA
22O53	Pampolat de Sagunto	wine	IMIDRA
22O55	Verdil	wine	IMIDRA
22O57	Grumiere Negro	wine	IMIDRA
22O66	Planta de Pedralba	wine	IMIDRA
25A01	Perla de Csaba	table	IMIDRA
25A04	Bruni 036	table	IMIDRA
25A05	Madeleine Royal	table	IMIDRA
25A11	Agostenga	wine	IMIDRA
25A15	Precoce de Malingre	table	IMIDRA
25A21	Muscat Saint Laurent	table	IMIDRA
25B05	El Gouz	table	IMIDRA
25B07	Gradiska	table	IMIDRA
25B08	Propseri 008	table	IMIDRA
25B10	Bruni 041	table	IMIDRA
25B11	Panse Precoce	table	IMIDRA
25B15	Admirable de Courtiller	table	IMIDRA
25B18	Angiola Bianca	table	IMIDRA
25B22	Palestina 1	table	IMIDRA
25C05	Foster's White Seedling	table	IMIDRA
25C12	Schiradzouli Bianco	table	IMIDRA
25C16	Meslier Hatif	table	IMIDRA
25C17	Diamant Traube	table	IMIDRA
25C19	Superzibibbo	table	IMIDRA
25C20	Japinkay	table	IMIDRA
25C21	Sauvignon	table	IMIDRA
25C23	Moscat Gustav Szauter	table	IMIDRA
25D01	Favorita de Cornegliano	table	IMIDRA
25D03	Wratchanski Misket	table	IMIDRA
25D05	Moscato Di Terracina	table	IMIDRA
25D08	Sideritis	table	IMIDRA
25D09	Aurora	table	IMIDRA
25D10	Zarina Na Losiata x Bolgar 19-2	table	IMIDRA
25D11	Lacrima Di Maria	table	IMIDRA
25D15	Ciclópica	table	IMIDRA

Code	Name	Main use	Repository
25D17	Baresana	table	IMIDRA
25E01	Smederevka	wine	IMIDRA
25E06	Gallura Zenni de Damasco	table	IMIDRA
25E07	Vivona 378	table	IMIDRA
25E12	Olivette Barthelet	table	IMIDRA
25E18	Pirovano 575	table	IMIDRA
25E22	Ciminnita	table	IMIDRA
25E24	Fogoneu	wine	IMIDRA
25F06	Talismano	table	IMIDRA
25F14	Volta	table	IMIDRA
25F16	Roi de Precoces	table	IMIDRA
25F17	Chaouch Rose	table	IMIDRA
25F19	Cardinal	table	IMIDRA
25F20	Perla de Csaba	table	IMIDRA
25F22	Chaselass Violet	table	IMIDRA
25F23	Chaselass Rose	table	IMIDRA
25G01	Portugues Azul	wine	IMIDRA
25G03	Queen Kearney	table	IMIDRA
25G14	Cornichon Violet	table	IMIDRA
25G17	Prune de Cazouls	table	IMIDRA
25G19	Alphonse Lavallee	table	IMIDRA
25G22	Apesorgia Nera	table	IMIDRA
25G23	Plovdina	wine	IMIDRA
25H02	Oillade Noir	table	IMIDRA
25H03	Piriquita	wine	IMIDRA
25H04	Moscato Del Adda	table	IMIDRA
25H05	Moscatel de Hamburgo	table	IMIDRA
25H07	Cinsaut	table	IMIDRA
25H10	Cilieguiolo Nero	table	IMIDRA
25H11	Criolla	table	IMIDRA
25H14	Superba	table	IMIDRA
25H18	Perla Nera	table	IMIDRA
25H19	Bruni 090	table	IMIDRA
25H22	Gemre	table	IMIDRA
25I02	Barbableu	table	IMIDRA
25I07	Pirovano 771	table	IMIDRA
25I08	Maria Rosa	table	IMIDRA
25I10	Inzolia Nera	table	IMIDRA
25I11	Darkaia Nera	table	IMIDRA
25I12	Royal	table	IMIDRA
25I15	Lassina	table	IMIDRA
25I16	Ahmeur Bou Ahmeur	table	IMIDRA
25I17	620 Pirovano	table	IMIDRA
25I18	Rosaki Noir des Semis	table	IMIDRA
25I19	Verico	table	IMIDRA
25I20	Fraula Kakini	table	IMIDRA
25I21	El Farryali	table	IMIDRA
25I22	Gros Maroc	table	IMIDRA
25I23	Danugue	table	IMIDRA
25I24	Mistress Hall	table	IMIDRA
25J11	Glaciere	table	IMIDRA
25J12	Prunesta Nera	wine	IMIDRA
25J13	Colgar Roja	table	IMIDRA
25J14	Pergolese	table	IMIDRA
25J15	Pizzutello Nero	table	IMIDRA
25J16	Almeria Nera	table	IMIDRA
25J17	Local Black	table	IMIDRA
25J18	Emperor	table	IMIDRA
25J19	Olivetta Nera	table	IMIDRA
25J23	Corinthe Blanc	table	IMIDRA
25J24	Chaselass Apyrene	table	IMIDRA
25J3	Black Alicante	table	IMIDRA
25J4	Dattier Noir	table	IMIDRA
25J7	Ophtalmo	table	IMIDRA
25J9	Marsigliana	wine	IMIDRA
25K03	Maria Pirovano	table	IMIDRA
25K09	Canner Seedless	table	IMIDRA
25K1	Graziella 1	table	IMIDRA
25K17	Black Currant	table	IMIDRA
25K19	Beauty Seedless	table	IMIDRA
25K2	Sultanina	table	IMIDRA
25K20	Black Monucca	table	IMIDRA
25K21	Sultanina Roja	table	IMIDRA
25K22	Sultanina Rosa	table	IMIDRA
25K4	Pirovano 166A	table	IMIDRA
25L03	Turki	table	IMIDRA
25L05	Conca D'oro	table	IMIDRA
25L20	Pirovano 155	table	IMIDRA
25L22	Zeini Abiad	table	IMIDRA
25M07	Al Izum Beli	table	IMIDRA
25M11	Balbal	table	IMIDRA
25M13	Patricia	table	IMIDRA
25M16	Marieta	table	IMIDRA
25VI_A03	Nincusa	table	IMIDRA
91_123_5	Dominga x Autumn Seedless 91-123-5	progeny	IMIDA[2]
91_88_29	Dominga x Autumn Seedless 91-88-29	progeny	IMIDA
91_88_31	Dominga x Autumn Seedless 91-88-31	progeny	IMIDA
91_88_77	Dominga x Autumn Seedless 91-88-77	progeny	IMIDA
91_89_10	Dominga x Autumn Seedless 91-89-10	progeny	IMIDA
91_89_13	Dominga x Autumn Seedless 91-89-13	progeny	IMIDA
91_89_32	Dominga x Autumn Seedless 91-89-32	progeny	IMIDA
91_90_01	Dominga x Autumn Seedless 91-90-01	progeny	IMIDA
91_90_04	Dominga x Autumn Seedless 91-90-04	progeny	IMIDA
91_90_05	Dominga x Autumn Seedless 91-90-05	progeny	IMIDA
91_90_10	Dominga x Autumn Seedless 91-90-10	progeny	IMIDA
91_90_11	Dominga x Autumn Seedless 91-90-11	progeny	IMIDA
91_90_12	Dominga x Autumn Seedless 91-90-12	progeny	IMIDA
91_90_15	Dominga x Autumn Seedless 91-90-15	progeny	IMIDA
91_90_18	Dominga x Autumn Seedless 91-90-18	progeny	IMIDA
Aut_S	Autumn Seedless	table	IMIDA
BA_1_6	Río Ardila	wild	IMIDRA
BA_2_1	Los Remedios 1	wild	IMIDRA
BA_2_2	Los Remedios 1	wild	IMIDRA
BA_2_3	Los Remedios 1	wild	IMIDRA
BA_2_4	Los Remedios 1	wild	IMIDRA
BA_2_5	Los Remedios 1	wild	IMIDRA
BA_2_6	Los Remedios	wild	IMIDRA
BA_2_6	Los Remedios	wild	IMIDRA
CA_10_1	Ribera de Barria	wild	IMIDRA
CA_11_1	La Algaida	wild	IMIDRA
CA_11_2	La Algaida	wild	IMIDRA

Code	Name	Main use	Repository
CA_11_3	La Algaida	wild	IMIDRA
CA_13_8	Arroyo Mayeruela	wild	IMIDRA
CA_2_7	Río Del Bosque	wild	IMIDRA
CA_3_4	Manatial El Quejido	wild	IMIDRA
CA_4_3	Pantano de Los Hurones	wild	IMIDRA
CA_5_1	Tio Tavizna	wild	IMIDRA
CA_6_1	Prado El Rey	wild	IMIDRA
CA_7_1	Las Montañas	wild	IMIDRA
CA_9_1	Ribera Millan	wild	IMIDRA
CO_1_1	Arroyo Bejarano	wild	IMIDRA
CO_1_2	Arroyo Bejarano	wild	IMIDRA
CO_1_4	Arroyo Bejarano	wild	IMIDRA
CO_1_5	Arroyo Bejarano	wild	IMIDRA
CO_1_6	Arroyo Bejarano	wild	IMIDRA
CO_1_7	Arroyo Bejarano	wild	IMIDRA
CO_1_8	Arroyo Bejarano	wild	IMIDRA
CO_2_1	Villaviciosa-Valdelasfuentes	wild	IMIDRA
CO_2_2	Villaviciosa-Valdelasfuentes	wild	IMIDRA
CO_2_4	Villaviciosa-Valdelasfuentes	wild	IMIDRA
CO_2_5	Villaviciosa-Valdelasfuentes	wild	IMIDRA
CO_2_6	Villaviciosa-Valdelasfuentes	wild	IMIDRA
CO_3_1	Posadas Río Guadalvacarejo	wild	IMIDRA
CO_3_2	Posadas Río Guadalvacarejo	wild	IMIDRA
CO_3_3	Posadas Río Guadalvacarejo	wild	IMIDRA
CO_3_4	Posadas Río Guadalvacarejo	wild	IMIDRA
CO_3_5	Posadas Río Guadalvacarejo	wild	IMIDRA
CO_3_6	Posadas Río Guadalvacarejo	wild	IMIDRA
CO_3_8	Posadas Río Guadalvacarejo	wild	IMIDRA
CO_4_1	Río Guadalquivir Alcolea	wild	IMIDRA
CO_4_2	Río Guadalquivir Alcolea	wild	IMIDRA
CO_4_3	Río Guadalquivir Alcolea	wild	IMIDRA
CO_4_5	Río Guadalquivir Alcolea	wild	IMIDRA
CO_4_8	Río Guadalquivir Alcolea	wild	IMIDRA
CO_4_9	Río Guadalquivir Alcolea	wild	IMIDRA
CO_5_1	Río Guadamellato	wild	IMIDRA
CO_5_2	Río Guadamellato	wild	IMIDRA
CO_5_4	Río Guadamellato	wild	IMIDRA
CO_5_5	Río Guadamellato	wild	IMIDRA
CO_6_1	Carretera de Cardeña	wild	IMIDRA
CO_6_2	Carretera de Cardeña	wild	IMIDRA
CO_6_3	Carretera de Cardeña	wild	IMIDRA
CO_6_4	Carretera de Cardeña	wild	IMIDRA
CO_6_5	Carretera de Cardeña	wild	IMIDRA
Crimson	Crimson	table	IMIDA
CSxM_10	Cabernet Sauvingon x Moscatuel 10	progeny	IMIDA
CSxM_16	Cabernet Sauvingon x Moscatuel 16	progeny	IMIDA
CSxM_19	Cabernet Sauvingon x Moscatuel 19	progeny	IMIDA
CSxM_23	Cabernet Sauvingon x Moscatuel 23	progeny	IMIDA
CSxM_24	Cabernet Sauvingon x Moscatuel 24	progeny	IMIDA
CSxM_27	Cabernet Sauvingon x Moscatuel 27	progeny	IMIDA
CSxM_30	Cabernet Sauvingon x Moscatuel 30	progeny	IMIDA
CSxM_32	Cabernet Sauvingon x Moscatuel 32	progeny	IMIDA
CSxM_33	Cabernet Sauvingon x Moscatuel 33	progeny	IMIDA
CSxM_35	Cabernet Sauvingon x Moscatuel 35	progeny	IMIDA
CSxM_39	Cabernet Sauvingon x Moscatuel 39	progeny	IMIDA
CSxM_40	Cabernet Sauvingon x Moscatuel 40	progeny	IMIDA
CSxM_41	Cabernet Sauvingon x Moscatuel 41	progeny	IMIDA
CSxM_5	Cabernet Sauvingon x Moscatuel 5	progeny	IMIDA
CSxM_7	Cabernet Sauvingon x Moscatuel 7	progeny	IMIDA
CSxM_8	Cabernet Sauvingon x Moscatuel 8	progeny	IMIDA
CS	Cabernet Sauvignon	wine	IMIDRA
Dominga	Dominga	table	IMIDA
GP_16	Garnacha Peluda Selfed 16	progeny	CNB[3]
GP_18	Garnacha Peluda Selfed 18	progeny	CNB
GP_4	Garnacha Peluda Selfed 4	progeny	CNB
GP_6	Garnacha Peluda Selfed 6	progeny	CNB
GP_8	Garnacha Peluda Selfed 8	progeny	CNB
GP_9	Garnacha Peluda Selfed 9	progeny	CNB
H_1_1	Rivera de Odiel	wild	IMIDRA
H_1_2	Rivera de Odiel	wild	IMIDRA
H_2_1	Río Murtiga	wild	IMIDRA
H_2_2	Río Murtiga	wild	IMIDRA
H_2_3	Río Murtiga	wild	IMIDRA
H_2_4	Río Murtiga	wild	IMIDRA
H_2_5	Río Murtiga	wild	IMIDRA
H_2_6	Río Murtiga	wild	IMIDRA
H_3_1	Barranco Olla	wild	IMIDRA
H_6_1	Marisma 1	wild	IMIDRA
H_6_3	Marisma 1	wild	IMIDRA
H_6_4	Marisma 1	wild	IMIDRA
H_7_2	Marisma 2	wild	IMIDRA
H_7_5	Marisma 2	wild	IMIDRA
MA_1_1	Venta El Hondon	wild	IMIDRA
MA_2_1	Río Turon 1	wild	IMIDRA
MA_2_3	Río Turon 1	wild	IMIDRA
MA_2_4	Río Turon 1	wild	IMIDRA
MA_2_7	Río Turon 1	wild	IMIDRA
MA_2_8	Río Turon 1	wild	IMIDRA
MA_3_1	Río Turon 2	wild	IMIDRA
MA_3_3	Río Turon 2	wild	IMIDRA
MA_3_4	Río Turon 2	wild	IMIDRA
MA_3_5	Río Turon 2	wild	IMIDRA
MA_3_6	Río Turon 2	wild	IMIDRA
MA_3_7	Río Turon 2	wild	IMIDRA
MA_3_8	Río Turon 2	wild	IMIDRA
MA_3_9	Río Turon 2	wild	IMIDRA
Monastrell	Monastrell	wine	IMIDA
Mos_Ham	Moscatel Hamburgo	table	IMIDA
MHxSO_23	Moscatel Hamburgo x Sugraone_23	progeny	IMIDA
MHxSO_24	Moscatel Hamburgo x Sugraone_24	progeny	IMIDA
MHxSO_25	Moscatel Hamburgo x Sugraone_25	progeny	IMIDA
MHxSO_26	Moscatel Hamburgo x Sugraone_26	progeny	IMIDA
MHxSO_27	Moscatel Hamburgo x Sugraone_27	progeny	IMIDA
MHxSO_28	Moscatel Hamburgo x Sugraone_28	progeny	IMIDA
MHxSO_29	Moscatel Hamburgo x Sugraone_29	progeny	IMIDA
MHxSO_33	Moscatel Hamburgo x Sugraone_33	progeny	IMIDA
MHxSO_34	Moscatel Hamburgo x Sugraone_34	progeny	IMIDA
MHxSO_35	Moscatel Hamburgo x Sugraone_35	progeny	IMIDA
MHxSO_36	Moscatel Hamburgo x Sugraone_36	progeny	IMIDA
Moscatu	Moscatuel	table	IMIDA
PT_1_1	Riveira de Toutalga	wild	IMIDRA

Code	Name	Main use	Repository
PT_1_5	Riveira de Toutalga	wild	IMIDRA
Ruby	Ruby Seedless	table	IMIDA
RxM_14	Ruby x Moscatuel 14	progeny	IMIDA
RxM_18	Ruby x Moscatuel 18	progeny	IMIDA
RxM_19	Ruby x Moscatuel 19	progeny	IMIDA
RxM_22	Ruby x Moscatuel 22	progeny	IMIDA
RxM_26	Ruby x Moscatuel 26	progeny	IMIDA
RxM_27	Ruby x Moscatuel 27	progeny	IMIDA
RxM_31	Ruby x Moscatuel 31	progeny	IMIDA
RxM_34	Ruby x Moscatuel 34	progeny	IMIDA
RxM_47	Ruby x Moscatuel 47	progeny	IMIDA
RxM_49	Ruby x Moscatuel 49	progeny	IMIDA
RxM_58	Ruby x Moscatuel 58	progeny	IMIDA
SE_1_1	Los Melonares	wild	IMIDRA
SE_1_2	Los Melonares	wild	IMIDRA
SE_2_1	Castillo de Las Guardas	wild	IMIDRA
SE_2_3	Castillo de Las Guardas	wild	IMIDRA
SE_2_4	Castillo de Las Guardas	wild	IMIDRA
SE_2_8	Castillo de Las Guardas	wild	IMIDRA
SE_3_1	El Martinete	wild	IMIDRA
SE_3_5	El Martinete	wild	IMIDRA
SE_4_1	Rivera del Huéznar	wild	IMIDRA
SNPs1	Cabernet Sauvignon	SNP discovery	IMIDRA
SNPs2	Syrah	SNP discovery	IMIDRA
SNPs3	Pinot Noir	SNP discovery	IMIDRA
SNPs4	Garnacha	SNP discovery	IMIDRA
SNPs5	Tempranillo	SNP discovery	IMIDRA
SNPs6	Malvasía de Sitges	SNP discovery	IMIDRA
SNPs7	Moscatel Grano Menudo	SNP discovery	IMIDRA
SNPs8	Sultanina	SNP discovery	IMIDRA
SNPs9	Ahmeur Bou Ahmeur	SNP discovery	IMIDRA
Sugraone	Sugraone	table	IMIDA
25_VII_A03	Torralba	wine	IMIDRA
25_VIII_B19	Bayad	table	IMIDRA
25_VIII_B20	Kischmisch Alí Blanc	table	IMIDRA

[1]IMIDRA: Instituto Madrileño de Investigación y Desarrollo Rural, Agrario y Alimentario

[2]IMIDA: Instituto Murciano de Investigación y Desarrollo Agrario y Alimentario

[3]CNB: Centro Nacional de Biotecnología
