# Supplementary figures and images for: High throughput SNP discovery and genotyping in grapevine (Vitis vinifera L.) by combining a re-sequencing approach and SNPlex technology
Source: BMC Genomics. 2007 Nov 19;8:424. doi: 10.1186/1471-2164-8-424 (PMC2212664; doi:10.1186/1471-2164-8-424)

MAF

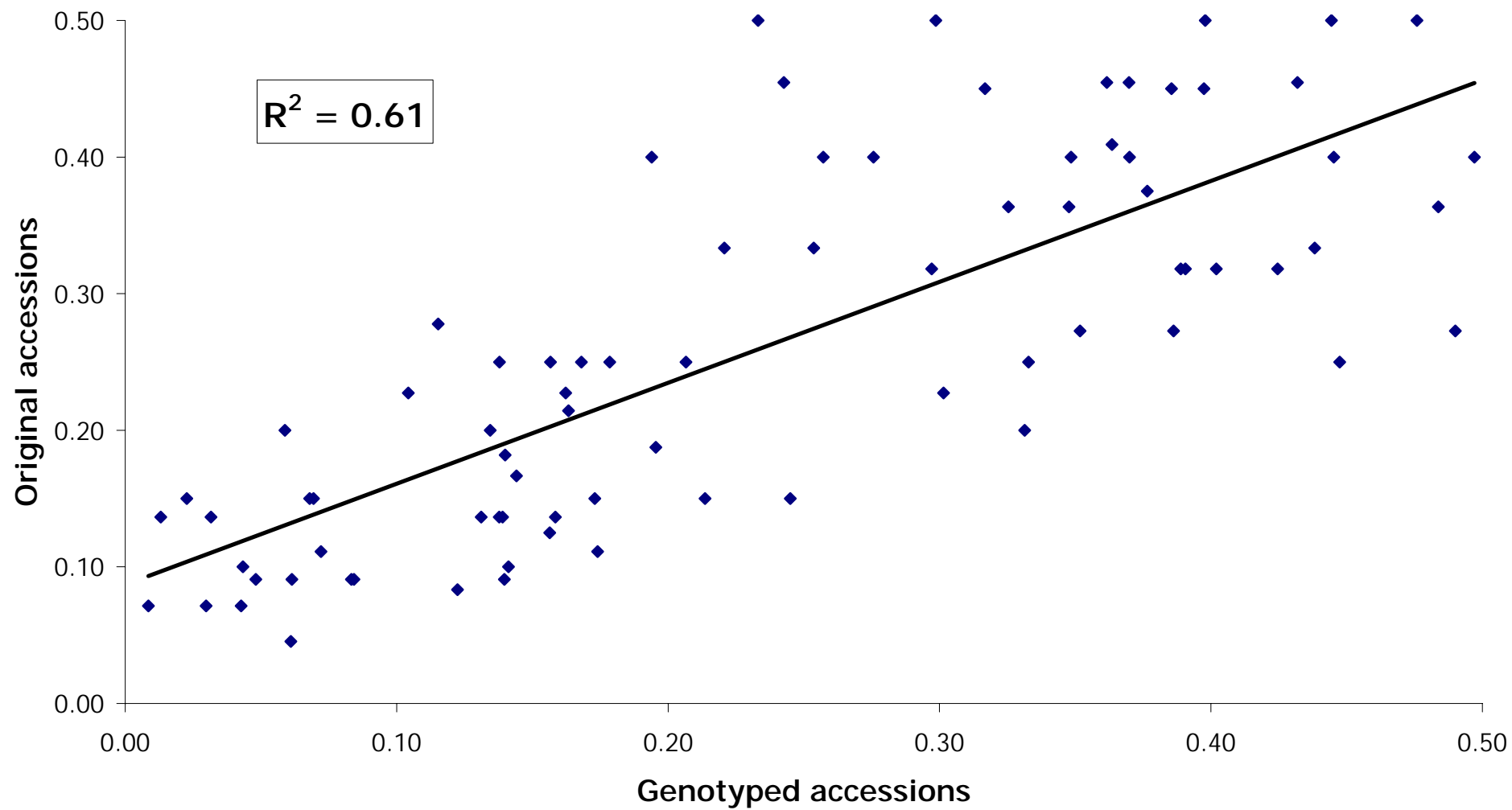

Supplement: Additional file 4 — Regression plot of MAF in genotyped accession vs. MAF in original accessions. PDF file displaying the linear regression between the MAF values for the 80 SNPs observed in the sample of ~300 genotyped accessions with the MAF values observed in the original sample of 11 genotypes use in the re-sequencing strategy. [file 1471-2164-8-424-S4.pdf]
