# Supplementary material for: High throughput SNP discovery and genotyping in grapevine (Vitis vinifera L.) by combining a re-sequencing approach and SNPlex technology
Source: BMC Genomics. 2007 Nov 19;8:424. doi: 10.1186/1471-2164-8-424 (PMC2212664; doi:10.1186/1471-2164-8-424)
Supplement: Additional file 5 — Global and class specific SNP minor allele frequencies. PDF file comparing the global minor allele frequency versus those observed in wild, table and wine classes for the 80 validated SNPs tested in the genotyping analysis. [file 1471-2164-8-424-S5.pdf]

SNP ID	All	Wild	Table	Wine
SNP1_351	0.17	**0.48**	**0.05**	**0.02**
SNP129_237	0.32	0.30	0.39	0.29
SNP135_316	0.26	**0.15**	**0.38**	**0.10**
SNP189_131	0.25	**0.11**	0.25	0.18
SNP191_100	0.17	**0.39**	0.11	0.08
SNP197_82	0.36	0.45	**0.27**	0.35
SNP209_255	0.18	**0.28**	0.14	0.13
SNP217_190	0.14	**0.27**	0.14	0.07
SNP227_191	0.30	**0.49**	**0.22**	0.22
SNP229_112	0.36	0.35	**0.27**	0.38
SNP241_201	0.21	0.24	0.15	**0.11**
SNP249_125	0.08	0.08	0.07	0.12
SNP255_265	0.37	**0.27**	**0.50**	0.26
SNP259_199	0.40	**0.23**	**0.49**	0.36
SNP269_308	0.50	**0.29**	**0.35**	**0.36**
SNP273_298	0.16	0.13	0.14	0.21
SNP273_361	0.06	0.02	0.08	0.05
SNP273_469	0.03	0.01	**0.07**	0.02
SNP281_64	0.33	0.37	0.29	0.40
SNP283_32	0.14	**0.05**	0.15	0.06
SNP289_84	0.33	**0.19**	0.28	**0.47**
SNP293_20	0.07	**0.01**	0.09	0.04
SNP311_198	0.42	**0.25**	**0.28**	**0.25**
SNP317_155	0.24	0.19	0.20	0.28
SNP325_65	0.20	**0.07**	0.19	**0.31**
SNP341_196	0.35	0.32	0.34	0.38
SNP345_421	0.04	0.06	0.05	0.04
SNP351_85	0.30	**0.10**	0.33	0.37
SNP355_154	0.44	0.39	0.37	**0.25**
SNP357_371	0.45	0.40	0.37	0.35
SNP377_251	0.37	0.40	0.39	0.29
SNP391_170	0.12	**0.21**	**0.06**	0.17
SNP397_331	0.13	**0.25**	0.10	0.08
SNP415_209	0.16	0.13	0.15	0.24
SNP421_234	0.13	**0.21**	0.07	0.18
SNP425_205	0.06	0.10	0.04	0.07
SNP437_129	0.19	**0.28**	0.16	**0.07**
SNP447_244	0.45	**0.33**	0.47	**0.28**
SNP451_287	0.35	0.32	0.34	0.38
SNP453_375	0.44	0.38	0.49	0.41
SNP457_192	0.04	**0.11**	0.03	0.01
SNP459_140	0.22	0.23	0.26	0.15
SNP463_296	0.02	**0.06**	0.01	0.01
SNP477_239	0.16	**0.41**	**0.06**	**0.06**
SNP497_281	0.07	0.03	0.09	0.06
SNP517_224	0.39	0.38	**0.29**	0.27
SNP533_161	0.01	0.00	0.02	0.00
SNP543_268	0.10	0.05	0.12	**0.20**
SNP551_351	0.12	0.06	0.11	**0.24**
SNP553_98	0.39	0.36	0.40	0.36
SNP555_132	0.38	0.40	0.31	**0.18**
SNP557_104	0.17	**0.08**	**0.28**	0.09
SNP559_110	0.23	0.22	0.25	0.24
SNP561_120	0.03	0.01	0.05	0.06
SNP567_341	0.21	0.24	0.18	**0.35**
SNP571_227	0.48	0.44	0.48	0.46
SNP575_128	0.14	0.11	0.13	**0.28**
SNP579_187	0.14	0.08	**0.08**	**0.30**
SNP581_114	0.39	**0.07**	**0.50**	0.47
SNP591_148	0.35	0.26	0.38	0.33
SNP593_149	0.39	0.35	0.46	0.28
SNP605_120i	0.14	**0.03**	0.21	0.15
SNP613_315	0.33	**0.46**	0.26	0.32
SNP625_278	0.08	**0.20**	**0.00**	0.04
SNP635_21	0.16	**0.25**	0.13	**0.05**
SNP649_567	0.40	0.38	**0.50**	0.32
SNP651_658	0.01	0.01	0.00	**0.05**
SNP653_90	0.30	**0.43**	0.23	**0.16**
SNP655_93	0.16	**0.31**	0.12	0.09
SNP659_73	0.39	0.45	0.41	0.31
SNP683_120	0.05	0.01	0.07	0.04
SNP691_139	0.49	0.38	0.48	0.49
SNP697_296	0.14	0.16	**0.07**	**0.30**
SNP699_311	0.14	0.16	0.21	0.08
SNP709_258	0.25	0.18	0.26	**0.40**
SNP715_260	0.06	0.02	0.07	0.07
SNP811_42	0.28	0.34	0.22	0.28
SNP817_209	0.07	0.07	0.06	0.07
SNP819_210	0.43	0.47	0.40	0.45
SNP829_281	0.48	0.35	0.49	0.48
